# lncEvo: automated identification and conservation study of long noncoding RNAs

**DOI:** 10.1186/s12859-021-03991-2

**Published:** 2021-02-09

**Authors:** Oleksii Bryzghalov, Izabela Makałowska, Michał Wojciech Szcześniak

**Affiliations:** Institute of Human Biology and Evolution, Faculty of Biology, Adam Mickiewicz University in Poznan, Uniwersytetu Poznanskiego 6, 61-614 Poznan, Poland

**Keywords:** lncRNAs, Orthologs, Synteny

## Abstract

**Background:**

Long noncoding RNAs represent a large class of transcripts with two common features: they exceed an arbitrary length threshold of 200 nt and are assumed to not encode proteins. Although a growing body of evidence indicates that the vast majority of lncRNAs are potentially nonfunctional, hundreds of them have already been revealed to perform essential gene regulatory functions or to be linked to a number of cellular processes, including those associated with the etiology of human diseases. To better understand the biology of lncRNAs, it is essential to perform a more in-depth study of their evolution. In contrast to protein-encoding transcripts, however, they do not show the strong sequence conservation that usually results from purifying selection; therefore, software that is typically used to resolve the evolutionary relationships of protein-encoding genes and transcripts is not applicable to the study of lncRNAs.

**Results:**

To tackle this issue, we developed lncEvo, a computational pipeline that consists of three modules: (1) transcriptome assembly from RNA-Seq data, (2) prediction of lncRNAs, and (3) conservation study—a genome-wide comparison of lncRNA transcriptomes between two species of interest, including search for orthologs. Importantly, one can choose to apply lncEvo solely for transcriptome assembly or lncRNA prediction, without calling the conservation-related part.

**Conclusions:**

lncEvo is an all-in-one tool built with the Nextflow framework, utilizing state-of-the-art software and algorithms with customizable trade-offs between speed and sensitivity, ease of use and built-in reporting functionalities. The source code of the pipeline is freely available for academic and nonacademic use under the MIT license at https://gitlab.com/spirit678/lncrna_conservation_nf.

## Background

Long noncoding RNAs (lncRNAs) represent a highly heterogeneous class of RNA molecules arbitrarily defined as transcripts of more than 200 nucleotides in length that are not translated into proteins. They are found in virtually all eukaryotes and are known to play essential biological roles. Despite growing efforts to understand the biology of lncRNAs, the origin, evolution and functions of the majority of lncRNAs remain unknown.

Evolutionary conservation has been proven to be a useful metric for evaluating the functional importance of genes, but the majority of lncRNAs are poorly conserved compared to protein-encoding genes [1]. According to Hezroni et al. [2], more than 70 percent of lincRNAs, i.e. autonomously transcribed lncRNAs that do not overlap annotated coding genes, cannot be linked to homologs in species that diverged > 50 million years ago. It should be noted, however, that there are diverse patterns of lncRNA conservation, which are believed to reflect their mode of action [3]. Most often, lncRNAs show only positional conservation, with the sequence itself showing little or no similarity to the assumed homologue (referred to as a syntenic homologue or syntolog) [4]. They are expected to exert their functions, such as cotranscriptional recruitment of the complex epigenetic machinery that mediates histone modifications, in a sequence-independent manner, leading to transcriptional regulation of genes in *cis* [5]. In contrast to syntenic transcripts, orthologous lncRNAs with high exon sequence identities are expected to play similar sequence-dependent roles in the two species of interest, and these functions could be exerted both in *cis* and in *trans* [6]. Finally, a number of lncRNAs show locus sequence identity with their homologues without preservation of the exonic sequences [2, 7]; this reflects scenarios in which only parts of the sequence are essential, such as motifs required for RNA:protein interactions or splicing signals [8, 9].

The lack of proper tools for integrative studies of lncRNAs that allow their identification from RNA-Seq data, the identification of orthologues and the characterization of their conservation properties, motivated us to develop a dedicated computational pipeline. The pipeline consists of our own scripts as well as a number of previously published software, which were carefully selected based on their performance, popularity and own experience. For example, reference-guided assembly of transcriptomes (also referred to as ab initio assembly), which takes advantage of a genome sequence to which RNA-Seq reads are aligned using splice-aware software, is done with StringTie [10]; the tool outperforms other software for transcriptome assembly, such as Cufflinks [11] and Bayesembler [12]. The identification of lncRNAs, on the other hand, benefits from a number of lncRNA features that are employed to differentiate mRNAs from noncoding RNAs [13]. For instance, Coding Potential Calculator [14], a part of our lncRNA search algorithm, is focused on the coding capability of transcripts and much relies on sequences of already known protein-coding genes from public databases.

By coupling lncRNA discovery and annotation with conservation studies, we ensured that the data required for interspecies analyses were prepared in a uniform manner and that annotation quality bias was minimized. The computational workflow, which is called lncEvo, uses raw RNA-Seq data as a starting point and returns a list of conserved long noncoding transcripts, allowing its users to obtain insights into the conservation characteristics of particular lncRNAs as well as ab initio assembled transcriptomes and sets of predicted lncRNAs, which can be outputted in several commonly used formats.

lncEvo represents a fully integrated automatic pipeline for transcriptome assembly and identification of lncRNAs, which can be followed by cross-species conservation analysis of noncoding transcriptomes. The pipeline offers ease of use with well-tested, optimized software packages with default settings; the user-inputted data are expected to be regular FASTQ files obtained from RNA-Seq experiments. The software is executed in a Docker container, providing flexibility to run lncEvo within a variety of infrastructures, while the *dataflow* programming model allows for straightforward and efficient parallelization of the computational tasks.

## Implementation

lncEvo is written using a reactive workflow framework called *Nexflow* [15]. *Nexflow* is based on the *dataflow* programming model, which greatly simplifies the coding of complex distributed pipelines with *Docker* containers [https://docker.com] as an executive environment. This means that the binary dependencies are contained within a standard and portable format and can be executed on any platform supporting the Docker engine. The *Conda* [https://conda.io] management system is applied to easily configure the workflow dependencies with *Conda* environment files.

### Docker container

The Nextflow script is transparently executed in a Docker container. There are two options: (1) to build the image from scratch using *Dockerfile* and the *docker build* command; (2) to use the *spirit678/lncrna_conservation:latest* repository from Docker Hub [https://hub.docker.com] (default option; may be changed in *docker.config*). As a base, *continuumio/miniconda3:4.7.12* is used with *procps* installed, while the list of software that is used is declared within the Conda environment files *environment.yml* and *cpc2.yml*. The applied Conda channels are *conda-forge*, *bioconda*, *khourhin*, *cbp44* and *defaults*.

### Input data

**Fig. 1:**
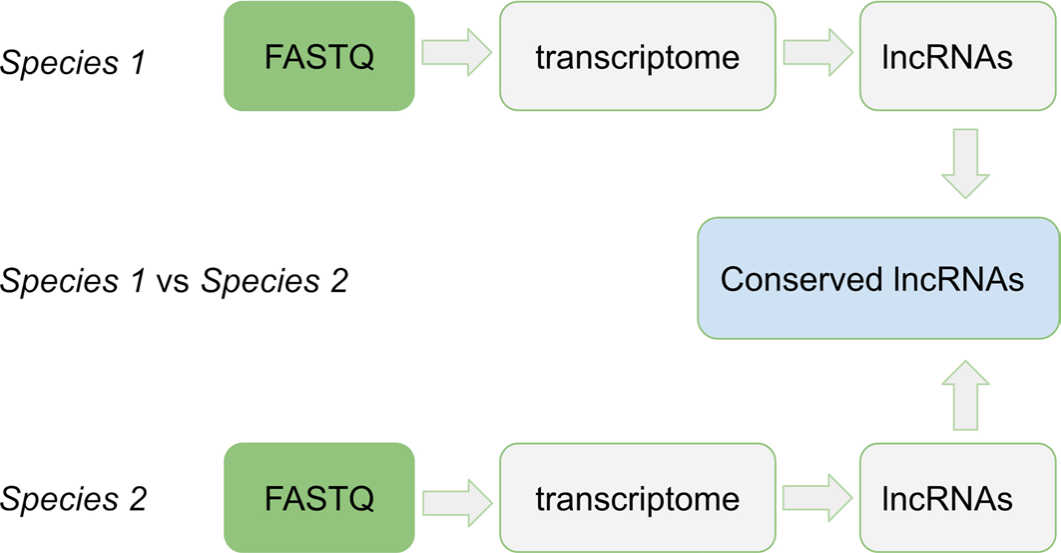
A schematic representation of the lncEvo workflow. There are two sequential parts: (1) ab initio transcriptome assembly from RNA-Seq data followed by identification of lncRNAs, and (2) the search for conserved counterparts in the two species of interest

As an input, the pipeline only requires paired-end FASTQ files and genome assembly IDs, such as GRCh38, Pan_tro_3.0, or the species’ names (here, human and chimpanzee, respectively) (Fig. 1). Currently, we support only species that are available in the ENSEMBL repository. A Python script is used to find a proper assembly version with ENSEMBL mart using a *pybiomart* library [https://jrderuiter.github.io/pybiomart/] to create the download queues and prepare additional datasets, if they are available (representing a set of ribosomal RNAs (rRNAs) and a set of known lncRNAs). The *requests*, *ftplib* and *urllib* Python libraries are used for downloading the sequences of rRNAs and toplevel FASTA files for a given genome assembly as well as the reference gene annotations in GTF format.

### Identification of lncRNAs

**Fig. 2:**
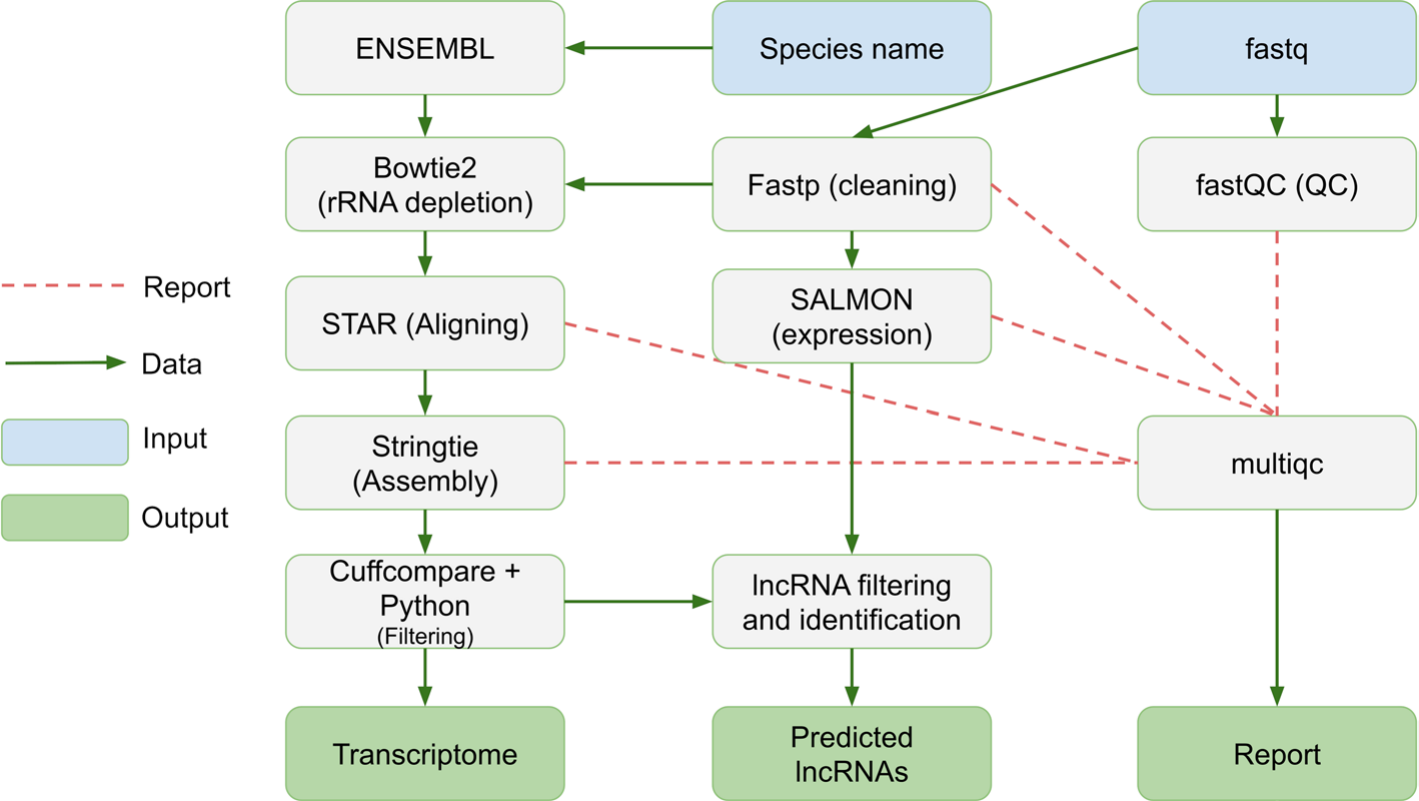
A schematic representation of the computational steps for ab initio transcriptome assembly and identification of lncRNAs. Data flow is represented with green arrows, whereas the metadata relationships are marked with red dotted lines. A more detailed representation of the workflow is available on the tool webpage

The identification task is divided into two parts: ab initio assembly of the transcriptome, which is followed by identification and filtering of the lncRNAs (Fig. 2). Starting with the FASTQ files, the initial quality check is performed using *FastQC* [http://www.bioinformatics.babraham.ac.uk/projects/fastqc/]. The metrics for this step and the other steps of transcriptome assembly are collected in a single HTML document with *multiQC* [16]. Quality filtering, trimming and clipping of the adapters is performed with either *fastp* [17] or *bbduk* [sourceforge.net/projects/bbmap/]. Fastp is used by default because of its unique combination of speed, quality and ease of use [17]. Subsequently, the rRNA-derived reads are discarded by mapping them against a set of ribosomal RNAs with *Bowtie 2* [18] and retaining only the unmapped reads. Then, mapping against the corresponding genome is performed with *STAR* [19] using the recommended settings [https://github.com/alexdobin/STAR/blob/master/doc/STARmanual.pdf]. For this, the genome index is built from a FASTA file and additional ENSEMBL annotations in GTF format to improve the accuracy of the splice mapping. The obtained BAM files (one per sample) represent the input for ab initio transcriptome assembly with *StringTie* [10] using annotations from ENSEMBL as a reference. The resulting GTF files with custom transcriptomes (one per sample) are merged with *StringTie* into a single transcriptome. The transcriptome is then compared against reference annotations with *Cuffcompare* [20], and transcripts belonging to the class codes *c*, *e*, *p* or *s*, which represent potential errors in transcriptome assembly, are removed. The sequences of transcripts are extracted from the corresponding genome with *gffread* [21]. The estimation of expression levels is performed using *Salmon* [22] and summarized with a custom Python script utilizing the *pandas* library.

The identification of lncRNAs consists of several filtering steps that are applied to the assembled transcriptome. In the first step, the transcriptome is compared against the reference annotations using *Cuffcompare* with the -R and -C options. Then, lncRNA identification is performed using a set of Python scripts according to the following criteria:

- Transcripts with Cuffcompare class codes = *j* or *o* are discarded if the reference gene is not classified as a lncRNA in ENSEMBL;
- Transcripts shorter than 200 bases are removed;
- Transcripts containing open reading frames (ORFs) identified by *TransDecoder* [23] with the *-m 100* (minimum protein length; default value) and *-S* (strand-specific) options are discarded;
- Transcripts classified as encoding proteins by the *Coding Potential Calculator *(*CPC*) [14] with the default settings are eliminated as well;
- Each transcript is required to meet the expression threshold of 1 TPM in at least one sample.

Regardless of the *TransDecoder* and *CPC* results, retained are all expressed RNAs classified as lncRNAs in ENSEMBL.

As a result, one obtains a set of lncRNAs in the GTF and FASTA formats as well as a tab-separated text file with the detailed annotation data. The overall lncRNA search procedure represents a modification of previously published protocols and was previously applied by us in large-scale studies [4, 24].

### Conservation study

**Fig. 3:**
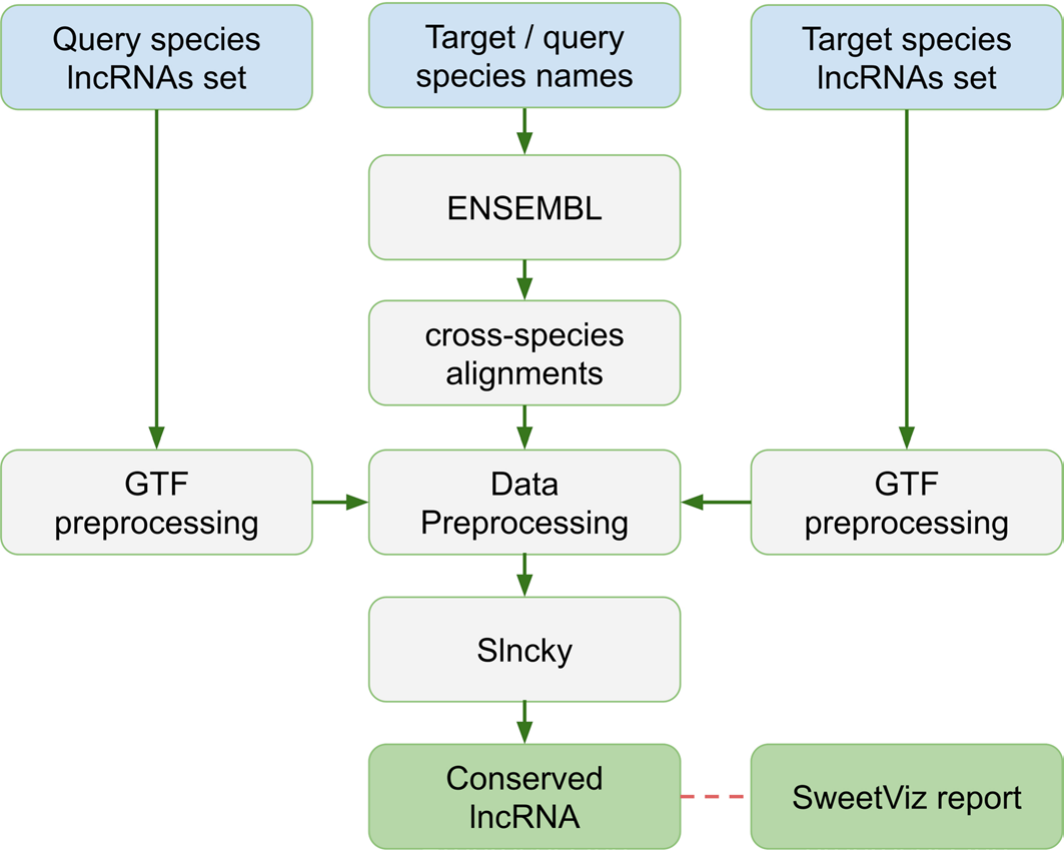
A schematic workflow for the lncEvo conservation study module. The module utilizes three consecutive steps: (1) preprocessing of the lncRNA annotations into the desired format, (2) preparation of the cross-species genome alignments, and (3) utilization of slncky for the pairwise conservation search, which is followed by postprocessing and generation of the final reports

Conservation analysis is performed in a pairwise manner between the two species of interest. The set of predicted lncRNAs in both species and the species names represent the input. One of the species is the *target*, providing the reference set of lncRNAs, while the *query* species is the source of potentially conserved counterparts (Fig. 3). A set of Python scripts is used to preprocess the data and build slncky-compatible annotations for the target and query transcriptomes.

Based on our previously described methodology [4], we redesigned the process of the generation of cross-species genome alignments, which allowed us to significantly reduce the required computational time while maintaining comparable sensitivity. The alignment itself is performed with *lastal* from the *LAST* package [25]. To prepare the genome index, we used the seeding scheme that corresponds to the estimated evolutionary distance; *NEAR* was used for closely related species (such as humans and chimps), and *MAM4* [26] was used in the remaining cases. Next, *last-train* [27] was used to determine the suitable substitution and gap scores for aligning the query and target sequences. The obtained matrix was then applied with *lastal* to produce the alignments in MAF format. As these steps are quite computationally intense, we conducted performance tuning to optimize the trade-offs among speed, sensitivity, memory and disk usage according to developer recommendations and our own observations. The resulting alignments were converted into a PSL format with the *maf-convert* [https://github.com/ENCODE-DCC/kentUtils] utility. Then, *axtChain* was applied with the *scoreScheme* produced by *last-train*, the genome sequences produced by the *faToTwoBit* utility and one of three distance options, “near”, “medium” or “far” (each determines the *minScore* and *linearGap* parameters). This produced a file with all chains of the alignments, which was then subject to chain netting with the *chainNet* and *netChainSubset* utilities and stored in the query species directory as a *target_species.query_species.over.chain.gz* file*.* Importantly, it is possible to use the chain files from UCSC if they are generated from the same genome versions; they should simply be placed in the root of the query species directory.In the next step, slncky [28] is used to identify the conserved counterparts of the query lncRNAs. The filtering options built into *slncky* are disabled, and only the orthology search is retained. A reference set of lncRNAs is divided into batches of 2500 transcripts for efficient computation, and each batch is processed independently. The results are merged into a single file with all orthologs and separately into a file with only the top-ranked orthologs to collect only the best query-target associations as determined by the calculated exonic identity of the two given lncRNAs.

Exploratory data analysis (EDA) is used to analyze the obtained data and provide the main characteristics of the datasets with the use of visual methods. To automate the EDA in the version of the pipeline designed for the local executor, summary reports are generated with a patched version of *SweetViz* [https://github.com/fbdesignpro/sweetviz]. The resulting HTML files provide visualizations as well as summary statistics for the predicted set of lncRNAs and the results of the conservation analysis. Additionally, comparison of the two sets of predicted lncRNAs or the results of the conservation analysis may be performed as an optional step.

### AWS batch implementation

**Fig. 4:**
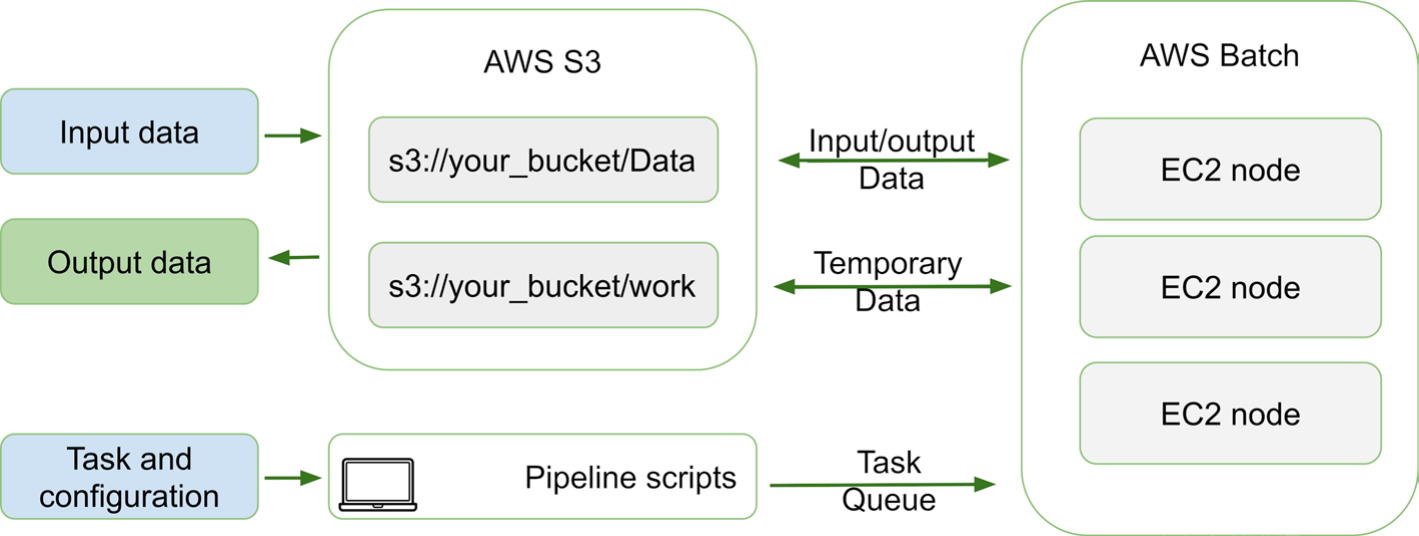
A schematic representation of AWS batch execution. A predefined S3 bucket is used as the pipeline working directory as well as the location used to store the input and output data. Execution scripts are located on the local machine, whereas the computational steps take place on the AWS batch cluster

In addition to the regular implementation of lncEvo, a separate execution script designed to be run in the cloud is provided (Fig. 4). The pipeline can be launched either on a local computer or as an *AWS EC2 instance*; the latter is suggested for heavy workloads or long runs.

*AWS Batch* dynamically provides the optimal quantity and type of computer resources based on the volume and specific resource requirements of the submitted batch jobs. Nextflow automatically manages the computing environment, allocates resources, and initiates and terminates nodes for each task according to the executed directed acyclic graph (DAG), job queues and computing environments. *AWS batch* settings are located within an *awsbatch.config* file. *Nextflow* retrieves the credentials from the user ~*/.aws/credentials* or ~*/.aws/config* files; alternatively, *AWS_ACCESS_KEY_ID* and *AWS_SECRET_ACCESS_KEY* may be specified in the environment.

The custom AMI should be based on the *ECS-Optimized Amazon Linux AMI* with an increased *ulimit* for open files (*-n 20,000*), an EBS volume of at least 500 GB on the Throughput Optimized HDD (st1), and an installed *AWS CLI* and Docker container size that is adjusted according to the EBS volume size. For testing, two computation environments were used—128 cores of m5.2xlarge (8 vCPUs; 32 GiB of RAM) for routine tasks and 128 cores of m5.8xlarge (32 vCPUs; 128 GiB of RAM) for intensive computations. Both types of instances utilized by Intel Xeon Platinum 8000 series processors.

### AWS Athena

*Athena* is an interactive query service that simplifies the analysis of data in *Amazon S3* using standard SQL. With *AWS Batch* implementation of the conservation analysis using an additional parameter, *–analytics yes*, a new folder named *Analytics* will be created in the Data directory of the *S3* bucket with the Hive-compatible partitioned data. The data schema is described in *Athena_schema.txt*. One can use Athena to run ad hoc queries with ANSI SQL without the need to aggregate or load the data into Athena. Athena integrates with Amazon *QuickSight* for easy data visualization. One can use Athena to generate reports or to explore data with business intelligence tools or SQL clients connected with a JDBC or an ODBC driver.

## Output

### The main output data

**Fig. 5:**
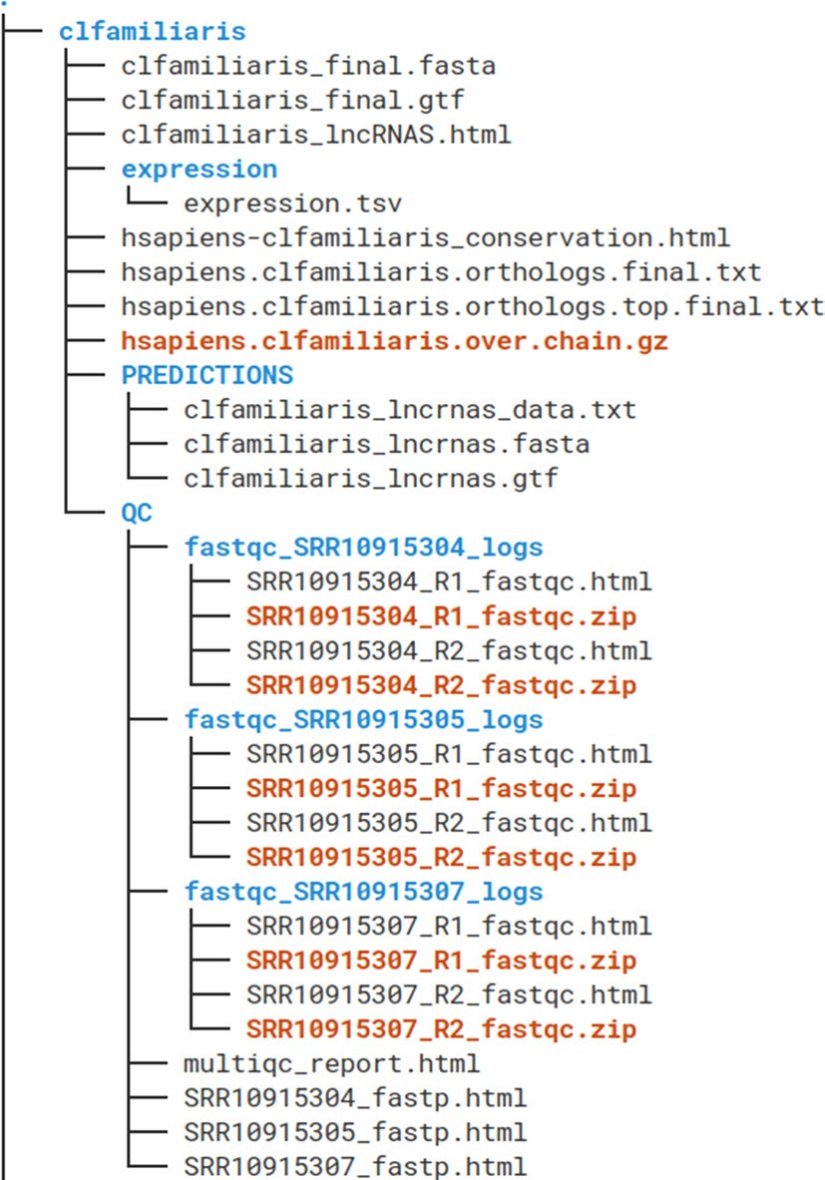
The structure of the output files, with an analysis of sequences from Canis lupus familiaris compared with human lncRNAs shown as an example. The QC folder contains multiple quality reports, with multiQC.html containing data from a number of software utilities. The set of predicted lncRNAs is kept in the PREDICTION folder, whereas the assembled transcriptome is available in the species_name_final.gtf and species_name_final.fasta files. The file hsapiens.clfamiliaris.over.chain.gz is the cross-species alignment chain file. Finally, the HTML files contain interactive reports

LncEvo generates output data for each of the three consecutive steps: transcriptome assembly, the lncRNA search and the lncRNA conservation analysis (Fig. 5). The transcriptome is available in GTF format for downstream processing and in a FASTA file, which contains the sequences of the transcripts. The transcript expression values can be found in a tab-separated file in the *expression* directory. The predicted lncRNAs are reported in GTF and FASTA formats as well, but there is also a TSV file with extended annotation data, such as the relationships of lncRNAs to the reference annotations, the results of the protein coding potential evaluation or the expression values provided in TPM units.

The output of the conservation study is presented in two files: one file contains the top-ranked orthologs selected based on the exonic identity, and the other file contains all of the possible conserved counterparts; the lncRNAs in both files are presented with the calculated conservation metrics. If a cross-species chain file is created, it is also stored for possible future runs using the same or other samples. Importantly, for lncRNA identification and conservation studies, interactive reports are provided in the form of HTML files that present the statistics in columns and the associations in rows. By implementing an additional analytic step (with a *-main comparison* flag), users can directly compare the summary statistics between the two noncoding transcriptomes or only the conserved counterparts.

### Quality reports and log files

Nextflow generates an HTML execution report with a number of metrics regarding workflow execution. The file is organized into three sections: *Summary*, *Resources* and *Tasks*. The *summary* section reports the execution status, launch commands, overall execution time and supplementary workflow metadata. The *Resources* section contains the plots of the distributions of resource usage for each workflow process, which are generated using the plotly.js JavaScript library. Finally, the *Tasks* section lists all executed tasks with their status and the actual command that was applied. There are also detailed reports for each of the lncEvo analytical steps. For transcriptome assembly, the *FastQC* HTML report provides the results of the quality control check of the raw input sequences; *Fastp* provides the quality control reports for the RNA-Seq data in a single HTML document. To aggregate the results of bioinformatics analyses of many samples and assembly stages into a single HTML report, *multiQC* is used.

## Performance and test run

To test lncEvo, five species were selected: human (*Homo sapiens*), mouse (Mus musculus), chimp (*Pan troglodytes*), horse (*Equus caballus*) and dog (*Canis familiaris*). In the conservation study, humans were used as a reference. The data used for testing were fetched from ENA using a Python script [https://github.com/wwood/ena-fast-download]. Data from the following Sequence Read Archive runs were analyzed: SRR4421334, SRR4421792, and SRR4421350 for humans; SRR7771840, SRR7771842, SRR7771843, and SRR7771846 for mice; SRR1602576, SRR1758919, and SRR1758927 for chimps; SRR9133801, SRR10140550, and SRR10205788 for horses; SRR10915304, SRR10915305, and SRR10915307 for dogs. Extensive details regarding analysis of human and mouse lncRNAs are provided in Additional file 1.

The computation time of the conservation process of the pipeline differed for different workflow executions and depended on the number of annotated lncRNAs in both species, the quality of the cross-species alignments and the evolutionary distance between the species (Table 1). Importantly, in the search for conserved lncRNAs, three distance options for building cross-species alignments are available: *near*, *medium* and *far* [https://github.com/mcfrith/last-genome-alignments]. The last two options may also be fine-tuned by using the *medium_fast* and *far_fast* versions, which in comparison with *medium* and *far* consume fewer resources and less CPU time but are expected to result in lower sensitivity. The *fast* options utilize the standard *-m10* flag for *Last* instead of *-m100* and enable the *-W99* parameter when building the *Last* index. Overall, it is recommended to use the *fast* options. Additional file 1 compares results obtained with medium and medium_fast parameters, with human lncRNAs used as a reference and a mouse as a target species. To reduce the computational time at the *slncky* stage, the target lncRNA dataset is divided into batches of 2500 transcripts each, which can be executed in parallel with the AWS Batch executor (one can use up to 10 forks at once).

**Table 1:** Transcriptome assembly and lncRNA identification test run metrics for selected species

Species	CPU-hours	Duration	Memory (max) (GB)
Human	134.1	6 h 14 m 47 s	39.07
Chimp	115.5	5 h 38 m 12 s	38.04
Horse	32.8	1 h 44 m 20 s	33.75
Dog	138.5	6 h 51 m 47 s	33.48
Mouse	115.3	5 h 28 m 17 s	36.47

One of the main advantages of lncEvo compared with the methodology used by us previously [4] is that lncEvo enables the parallelization of tasks. For transcriptome assembly using AWS Batch, the pipeline can handle as many samples at once as the cluster capacity allows, whereas for local execution, this is true only for tasks that do not utilize all available CPUs; however, it still represents a meaningful improvement for the pipeline.

### Results of the test runs

**Fig. 6:**
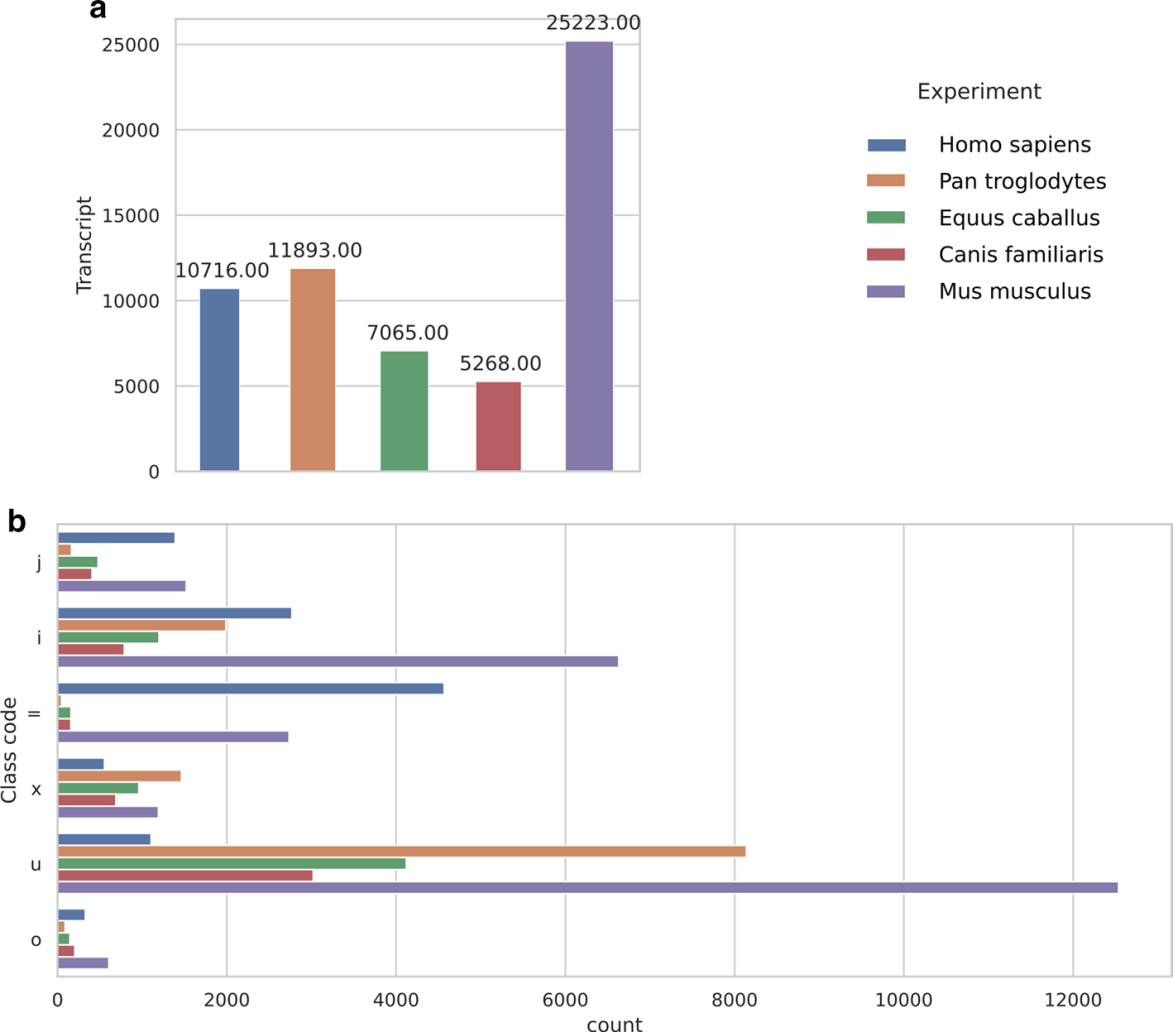
**a** lncRNAs found in each of the four species in test runs of lncEvo; **b** class codes of lncRNA transcripts identified according to Cuffcompare in comparison to those identified based on the reference ENSEMBL annotations; “=”: complete match with the intron chain; “j”: potential novel isoform; “i”: transcript falls entirely within a reference intron; “o”: generic exonic overlap with a reference transcript; “u”: unknown intergenic transcript; “x”: exonic overlap with a reference transcript on the opposite strand. The distributions reflect the quality of the ENSEMBL annotations, but the volume of the sequencing data and the types of samples used also have an impact on the distributions (some tissues possess a much more diverse repertoire of lncRNAs than other tissues)

Using the tested datasets, we predicted from ~ 5000 lncRNA transcripts in dogs up to ~ 25,000 in mice (Fig. 6). The differences across species may be explained by the quality of the available annotations and the number and sources of samples used in analysis (i.e., samples from diverse types of tissues, organs and cell lines). For humans, most of the lncRNAs fall into one of the three *Cuffcompare* class codes: “ = ” (equal to known ENSEMBL transcript), “i” (intronic variants of known genes) and “j” (novel isoforms of known genes) (Fig. 6). In the other species, most of the transcripts represent novel intergenic transcripts (class code “u”); this reflects the fact that most species have poor lncRNA annotations, which justifies our strategy of identifying the lncRNAs ourselves rather than relying on publicly available datasets.

**Fig. 7:**
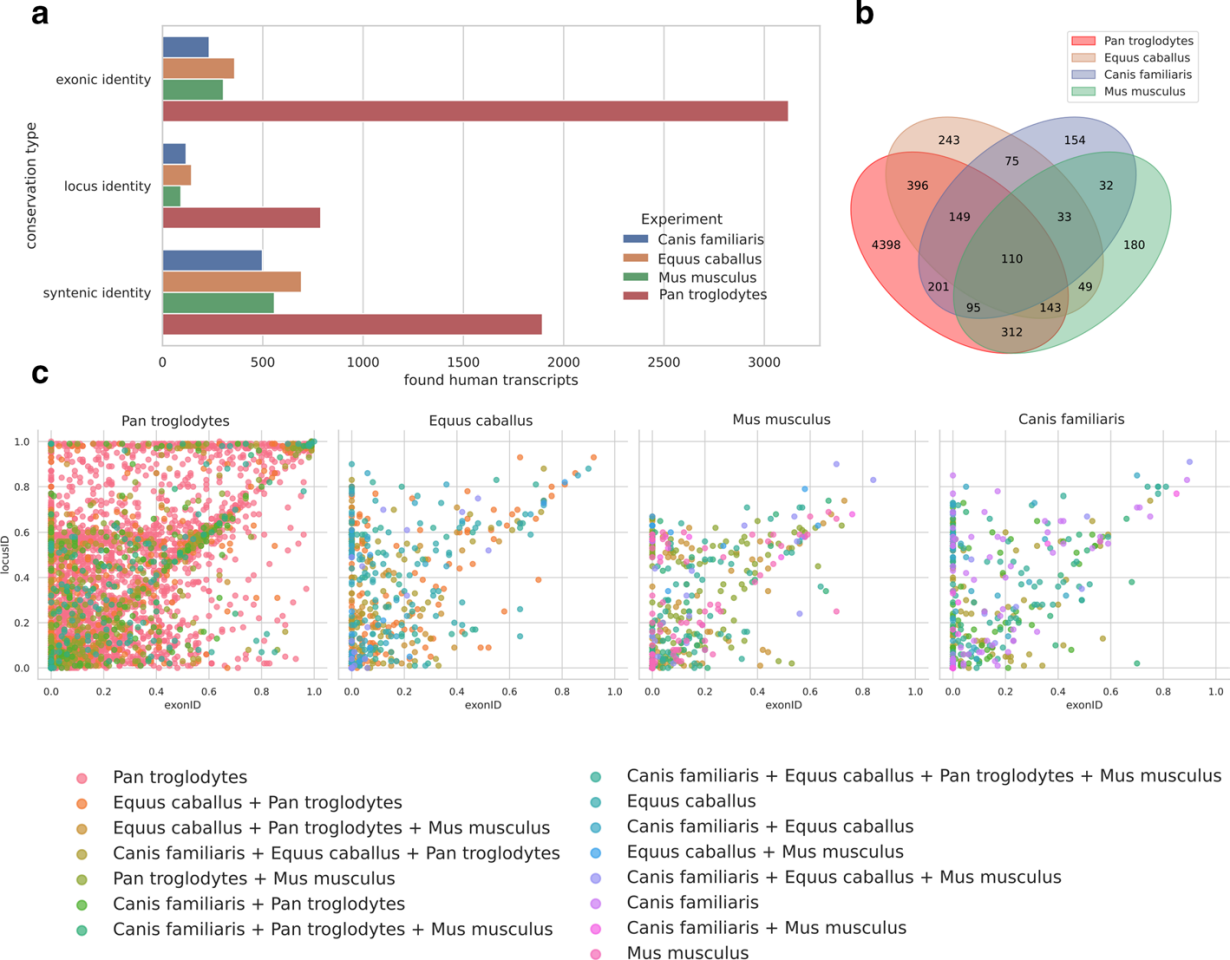
**a** Identified conserved counterparts of human lncRNA transcripts in four species classified according to the type of sequence conservation. Positional identity—the transcripts display only positional conservation; locus identity—significant sequence similarity in transcript-gene alignment detected; exonic identity—besides locus identity, there is also detectable sequence similarity in a transcript-transcript alignment. **b** The extent of the overlap with conserved human lncRNAs in the four analyzed species. **c** The color of the dots represents the species in which a given human transcript is conserved. The exonic identity is represented by the X axis, while locus identity is shown on the Y axis

In the conservation study, humans were used as the reference species during the cross-species analysis of mice, chimps, horses and dogs. The conserved transcripts were automatically assigned to one of three categories based on the extent of the sequence conservation (Fig. 7a); in mice, horses and dogs, half of the transcripts represent only the syntenic identity, while the conserved counterparts in chimps typically show high exonic identity with transcripts in humans, which is easily explained by the relatively short evolutionary distance between the two species. Interestingly, more than half of the lncRNAs conserved in mice, horses and dogs were also detected in the human-chimp comparison (Fig. 7b). On the other hand, the majority of transcripts conserved in the chimp were not detected in the other species, thus hinting that they might represent lineage-specific lncRNAs (Fig. 7c).

We were interested to see how switching the species affects the obtained results. To this point, we performed the analysis with human (query) vs mouse and, separately, mouse (query) vs human. As expected, the sets of obtained conserved lncRNAs were not identical but differences were not critical. For example, there were 550 positionally conserved lncRNAs for a human-mouse pair and 569 for the second one (see Table 8 in Additional file 1 for more details). The differences stem from a couple of factors, such as the size and completeness of the compared transcriptomes (the human and mouse sets of lncRNAs are summarized in Additional file 1). Another reason is that a syntenic region is going to be different in human-mouse than in mouse-human comparison, which is due to differences in genomic sequences and the available annotations, including protein-coding genes. Once a transcript is detected in a syntenic region, the cross-species alignment is scored relative to a set of random intergenic regions from one of the genomes—the score value (hence a decision whether a lncRNA is conserved) is going to be different, depending on which species is used as a query.

Finally, we checked the performance of lncEvo on a manually selected set of eleven lncRNAs, whose evolutionary conservation has been previously studied. For that purpose, we looked into human lncRNAs and their conserved counterparts in mice (Additional file 1). The analysis demonstrates lncEvo is able to efficiently detect lncRNAs conserved between humans and mice, such as MALAT1 or GAS5, often yielding multiple splicing isoforms of the lncRNA gene. As expected, it finds no orthologs for a primate-specific lncRNA (LINC00473).

### Downstream analyses

LncEvo produces a diversity of tabular data that can be fed into analytical platforms, thus enabling to query, filter the data, and plot graphs, among others. For users who deal with a number of species or transcriptomes and want to track a specific gene, transcript or a group of transcripts, such as coexpressed transcripts or ultraconserved lncRNAs, we have implemented Athena with AWS, thus providing a relatively straightforward way to manipulate the data using SQL syntax, without the requirement to build the actual relational database. Athena is also fully integrated with AWS QuickSight for data visualizations. On the other hand, for those wishing to obtain insight into the overall data statistics, including advanced plotting, SweetViz reports are a feasible option.

## Conclusions

Here, we present the first fully automated toolbox for the discovery and conservation study of mammalian noncoding transcripts based on raw RNA-Seq data. We made the process reproducible by using a stable software stack with tuned and predefined settings. We believe that the prepossessing and generation of the noncoding transcriptomes is an essential step for studies involving more than one species. We provide options to optimize the trade-off between speed and sensitivity as well as the freedom to choose the computational infrastructure. Additionally, we focused on extensive quality control and provided options for downstream analysis, such as AWS Athena. For brief and convenient comparisons of the obtained results, SweetViz reports are generated.

### Availability and requirements

Project name: lncEvo: automated identification and conservation study of long noncoding RNAs

Project home page: https://gitlab.com/spirit678/lncrna_conservation_nf

Operating system(s): platform independent

Programming language: Nextflow DSL

Other requirements: >= Java 8, >= Nextflow 2.20, >= Docker 19.03

License: MIT

Any restrictions to use by non-academics: none

## Supplementary Information

**Additional file 1**. An example of exploratory data analysis performed using lncEvo. It contains four sections: i) characteristics of used datasets, ii) a summary for lncRNA conservation analyses, iii) comparison of results obtained with different lncEvo settings, iv) case studies.

## Data Availability

Source code and the most recent version of lncEvo are freely available under MIT license in the GitLab repository: https://gitlab.com/spirit678/lncrna_conservation_nf. An archived version, referenced in the manuscript, is available at https://doi.org/10.5281/zenodo.4228473 as well as at https://gitlab.com/spirit678/lncrna_conservation_nf/-/releases/1.0. A Docker image is available at Docker Hub: https://hub.docker.com/repository/docker/spirit678/lncrna_conservation. Example result data from our test runs (predicted lncRNAs and their conserved counterparts in another species) are available on AWS s3: https://diffsuff.s3.amazonaws.com/for_paper/test_run_data.tar.gz. Additionally, interactive summary reports for the test runs can be obtained from https://diffsuff.s3.amazonaws.com/for_paper/test_run_reports.tar.gz.

## References

[1] Johnsson P, Lipovich L, Grandér D, Morris KV (2014). Evolutionary conservation of long non-coding RNAs; sequence, structure, function. Biochim Biophys Acta.

[2] Hezroni H, Koppstein D, Schwartz MG, Avrutin A, Bartel DP, Ulitsky I (2015). Principles of long noncoding RNA evolution derived from direct comparison of transcriptomes in 17 species. Cell Rep.

[3] Perry RB, Ulitsky I (2016). The functions of long noncoding RNAs in development and stem cells. Development.

[4] Bryzghalov O, Szcześniak MW, Makałowska I (2020). SyntDB: defining orthologues of human long noncoding RNAs across primates. Nucleic Acids Res.

[5] Engreitz JM, Haines JE, Perez EM, Munson G, Chen J, Kane M, McDonel PE, Guttman M, Lander ES (2016). Local regulation of gene expression by lncRNA promoters, transcription and splicing. Nature.

[6] Ruiz-Orera J, Mar AM (2019). Conserved regions in long non-coding RNAs contain abundant translation and protein–RNA interaction signatures. NAR Genomics Bioinform.

[7] Ulitsky I (2016). Evolution to the rescue: using comparative genomics to understand long non-coding RNAs. Nat Rev Genet.

[8] Schüler A, Ghanbarian AT, Hurst LD (2014). Purifying selection on splice-related motifs, not expression level nor RNA folding, explains nearly all constraint on human lincRNAs. Mol Biol Evol.

[9] Haerty W, Ponting CP (2015). Unexpected selection to retain high GC content and splicing enhancers within exons of multiexonic lncRNA loci. RNA.

[10] Pertea M, Pertea GM, Antonescu CM, Chang TC, Mendell JT, Salzberg SL (2015). StringTie enables improved reconstruction of a transcriptome from RNA-seq reads. Nat Biotechnol..

[11] Trapnell C, Roberts A, Goff L, Pertea G, Kim D, Kelley DR, Pimentel H, Salzberg SL, Rinn JL, Pachter L (2012). Differential gene and transcript expression analysis of RNA-seq experiments with TopHat and Cufflinks. Nat Protoc.

[12] Maretty L, Sibbesen JA, Krogh A (2014). Bayesian transcriptome assembly. Genome Biol.

[13] Li J, Zhang X, Liu C (2020). The computational approaches of lncRNA identification based on coding potential: status quo and challenges. Comput Struct Biotechnol J.

[14] Kang YJ, Yang DC, Kong L, Hou M, Meng YQ, Wei L, Gao G (2017). CPC2: a fast and accurate coding potential calculator based on sequence intrinsic features. Nucleic Acids Res..

[15] Di Tommaso P, Chatzou M, Floden EW, Barja PP, Palumbo E, Notredame C (2017). Nextflow enables reproducible computational workflows. Nat Biotechnol.

[16] Ewels P, Magnusson M, Lundin S, Käller M (2016). MultiQC: summarize analysis results for multiple tools and samples in a single report. Bioinformatics.

[17] Chen S, Zhou Y, Chen Y, Gu J (2018). fastp: an ultra-fast all-in-one FASTQ preprocessor. Bioinformatics.

[18] Langmead B, Salzberg SL (2012). Fast gapped-read alignment with Bowtie 2. Nat Methods.

[19] Dobin A, Davis CA, Schlesinger F, Drenkow J, Zaleski C, Jha S, Batut P, Chaisson M, Gingeras TR (2013). STAR: ultrafast universal RNA-seq aligner. Bioinformatics.

[20] Trapnell C, Williams BA, Pertea G, Mortazavi A, Kwan G, van Baren MJ, Salzberg SL, Wold BJ, Pachter L (2010). Transcript assembly and quantification by RNA-Seq reveals unannotated transcripts and isoform switching during cell differentiation. Nat Biotechnol.

[21] Pertea G, Pertea M. GFF utilities: GffRead and GffCompare. F1000Res. 2020;9:ISCB Comm J-304. 10.12688/f1000research.23297.1 PMC7222033 32489650

[22] Patro R, Duggal G, Love MI, Irizarry RA, Kingsford C (2017). Salmon provides fast and bias-aware quantification of transcript expression. Nat Methods.

[23] Haas BJ, Papanicolaou A, Yassour M, Grabherr M, Blood PD, Bowden J, Couger MB, Eccles D, Li B, Lieber M, MacManes MD, Ott M, Orvis J, Pochet N, Strozzi F, Weeks N, Westerman R, William T, Dewey CN, Henschel R, LeDuc RD, Friedman N, Regev A (2013). De novo transcript sequence reconstruction from RNA-seq using the Trinity platform for reference generation and analysis. Nat Protoc.

[24] Szcześniak MW, Wanowska E, Mukherjee N, Ohler U, Makałowska I (2020). Towards a deeper annotation of human lncRNAs. Biochim Biophys Acta Gene Regul Mech.

[25] Kiełbasa SM, Wan R, Sato K, Horton P, Frith MC (2011). Adaptive seeds tame genomic sequence comparison. Genome Res.

[26] Frith MC, Noé L (2014). Improved search heuristics find 20,000 new alignments between human and mouse genomes. Nucleic Acids Res.

[27] Hamada M, Ono Y, Asai K, Frith MC (2017). Training alignment parameters for arbitrary sequencers with LAST-TRAIN. Bioinformatics.

[28] Chen J, Shishkin AA, Zhu X, Kadri S, Maza I, Guttman M, Hanna JH, Regev A, Garber M (2016). Evolutionary analysis across mammals reveals distinct classes of long non-coding RNAs. Genome Biol.

