# Supplementary material for: lncEvo: automated identification and conservation study of long noncoding RNAs
Source: BMC Bioinformatics. 2021 Feb 9;22:59. doi: 10.1186/s12859-021-03991-2 (PMC7871587; doi:10.1186/s12859-021-03991-2)
Supplement: Supplementary file 1 — Additional file 1. An example of exploratory data analysis performed using lncEvo. It contains four sections: i) characteristics of used datasets, ii) a summary for lncRNA conservation analyses, iii) comparison of results obtained with different lncEvo settings, iv) case studies. [file 12859_2021_3991_MOESM1_ESM.pdf]

lncEvo: an example of Exploratory Data Analysis

January 7, 2021

Contents

1 lncRNA sets **2**
- 1.1 Data used 2
- 1.2 Human transcriptome 2
- 1.3 Mouse transcriptome 3
- 1.4 Comparison of Mouse and Human transcriptomes 4

2 Conservation **5**
- 2.1 Human transcripts conserved in Mouse (medium distance, lower sensitivity, computationally lite) 5
  - 2.1.1 Transcripts with highest identity 7
  - 2.1.2 Transcript-Transcript identity (exonID) 8
  - 2.1.3 Transcript-Locus identity (locusID) 8
- 2.2 Human transcripts conserved in Mouse (medium distance, high sensitivity, computationally expensive) 9
  - 2.2.1 Transcripts with highest identity 13
  - 2.2.2 Transcript-Transcript identity (exonID) 14
  - 2.2.3 Transcript-Locus identity (locusID) 14
- 2.3 Mouse transcripts conserved in Human 15
  - 2.3.1 Transcripts with highest identity 18
  - 2.3.2 Transcript-Transcript identity (exonID) 18
  - 2.3.3 Transcript-Locus identity (locusID) 19

3 Comparison of obtained datasets **20**
- 3.1 Comparison of pairs human-mouse and mouse-human 20
  - 3.1.1 Level of transcript pairs human-mouse and mouse-human 20
  - 3.1.2 Level of data-sets 23
  - 3.1.3 Summary 25
- 3.2 Comparison of human-mouse datasets (fast vs sensitive options) 25
  - 3.2.1 Genearal 25
  - 3.2.2 Comparison of human transcript biotypes 26
  - 3.2.3 Comparison conservation properties, regarding to transcript biotype 26
  - 3.2.4 Summary 29

4 Case study **29**
- 4.1 Growth arrest-specific 5 RNA (GAS5) 29
- 4.2 MALAT1 30

4.3 HOTAIRM1 31
4.4 The X inactive specific transcript 32
4.5 CHD2 adjacent, suppressive regulatory RNA(CHASERR) 32
4.6 CEROX1 cytoplasmic endogenous regulator of oxidative phosphorylation 1 33
4.7 LINC00473 (PDE10A) (primate specific) 33
4.8 FTX (FTX transcript, XIST regulator) 34
4.9 JPX (JPX, XIST activator) 35
4.10 RMST (Rhabdomyosarcoma 2 Associated Transcript) 35
4.11 ADAMTS9-AS2 36

1 lncRNA sets

1.1 Data used

Human Data Data from the following Sequence Read Archive runs were analyzed: SRR4421334, SRR4421792, and SRR4421350.

Mouse Data Data from the following Sequence Read Archive runs were analyzed: SRR7771840, SRR7771842, SRR7771843 and SRR7771846.

1.2 Human transcriptome

```
Shape of Human lncRNA dataset  (10716, 28)
```

```
No duplicates found
```

```
4568 out of 10716 human transcripts are equal to ENSEMBL transcripts, and
belongs to 3952 ENSEMBL genes
```

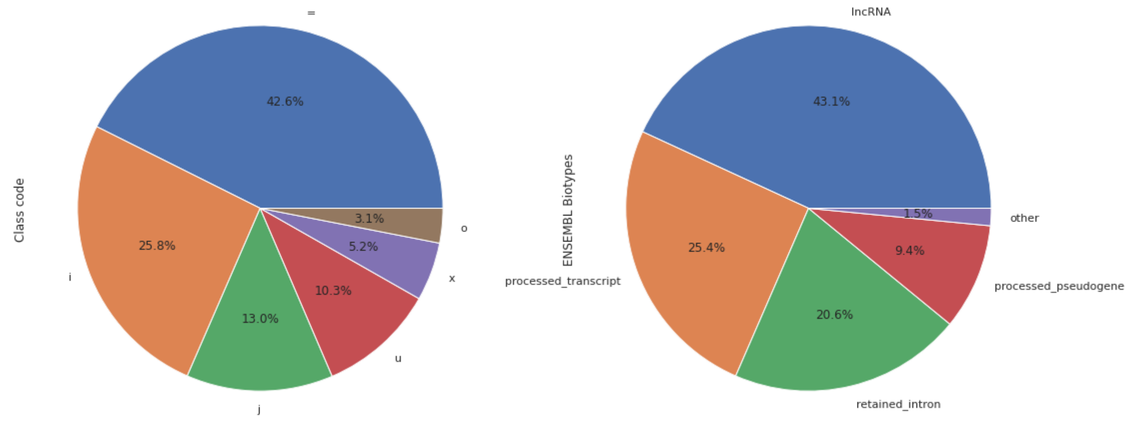

Figure 1. **(A)** Cufflinks classcodes for all noncoding transcripts in found in Human on the stage of transcriptome assembly. **(B)** Biotypes for transcripts, which are equal to ENSEMBL transcripts, e.g. for classcode "=".

Reference http://cole-trapnell-lab.github.io/cufflinks/cuffcompare/

<Figure size 1440x720 with 0 Axes>

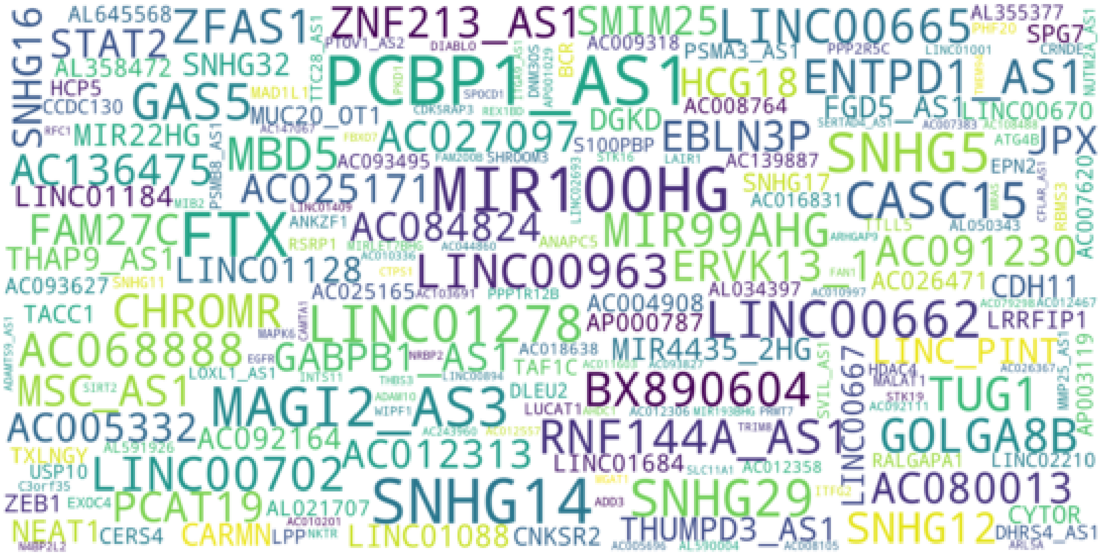

Figure 2. 200 most frequent Gene Names corresponded to noncoding transcripts, with classcode equal to ensembl

1.3 Mouse transcriptome

```
Shape of Mouse lncRNA dataset  (25223, 28)
```

```
No duplicates found
```

```
2736 out of 25223 human transcripts are equal to ENSEMBL transcripts, and
belongs to 2489 ENSEMBL genes
```

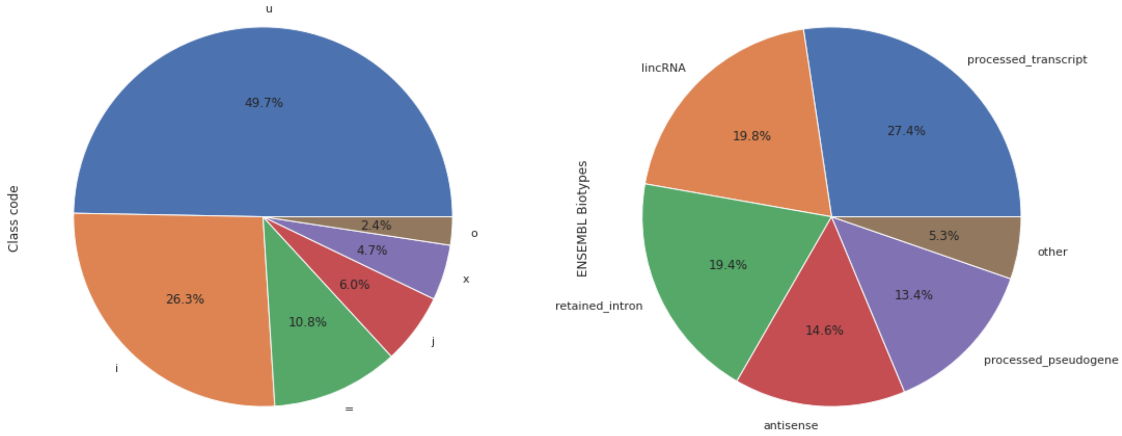

Figure 3. **A** Cufflinks classcodes for all noncoding transcripts found in Mouse on the stage of transcriptome assembly. **B** Biotypes for transcripts, which are equal to ENSEMBL transcripts, e.g. for classcode “=”.

Reference http://cole-trapnell-lab.github.io/cufflinks/cuffcompare/

```
<Figure size 1440x720 with 0 Axes>
```

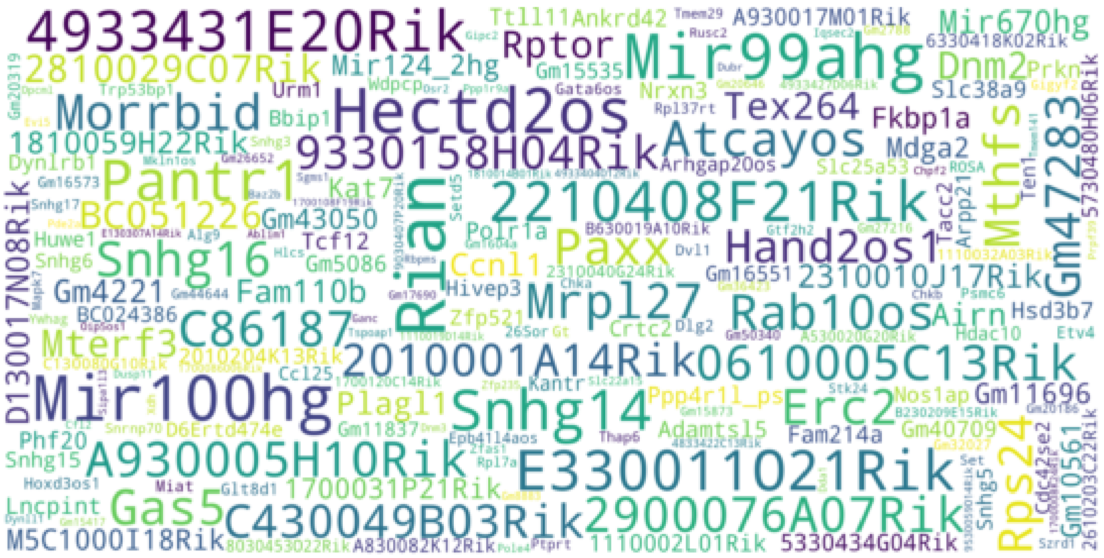

Figure 4. 200 most frequent Gene Names corresponded to non-coding transcripts, with classcode equal to ensembl Link to end of file

1.4 Comparison of Mouse and Human transcriptomes

In general the quality of assembled transcriptomes are highly dependent on the type of input data. We keep only lncRNAs passed our filters, including expression threshold. From our experience, more libraries users use - more complete transcriptomes are built. The difference between transcriptome is more flatten with adding each additional library into analysis.

[16]:

	Human	Mouse	Difference
Feature			
Total	10716	25223	14507
Transcripts equal to ENSEMBL (transcripts)	4568	2736	1832
Transcripts equal to ENSEMBL (genes)	3952	2489	1463
Novel intergenic lncRNAs	1106	12537	11431
Different isoforms of known genes (transcripts)	4488	8758	4270
Different isoforms of known genes (genes)	3172	8758	5586
ansinsense transcript(classcode ’x’)	554	1192	638

2 Conservation

2.1 Human transcripts conserved in Mouse (medium distance, lower sensitivity, computationally lite)

```
Shape of top final conserved human lncRNAs in Mouse  (942, 15)
```

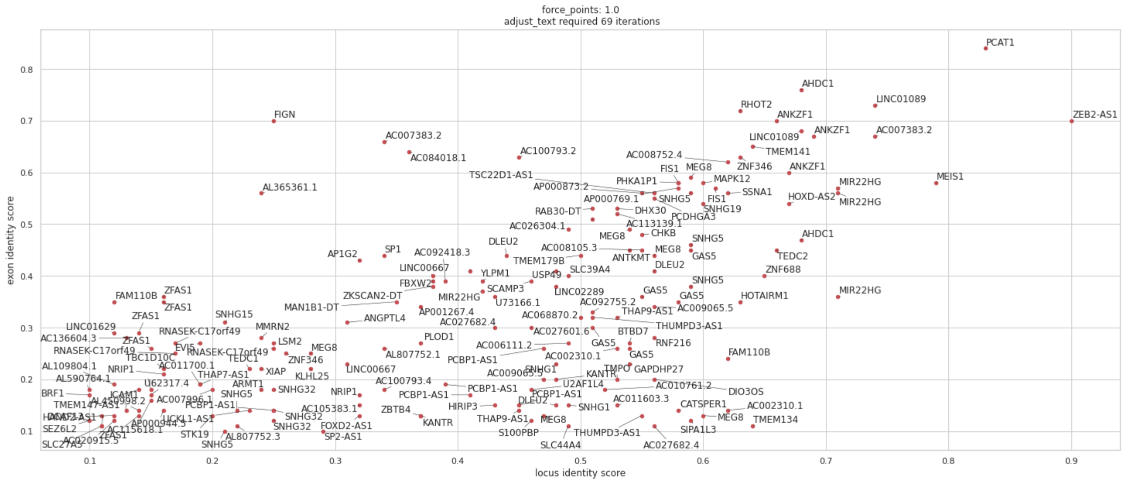

Figure 5. Human genes, with transcripts, showed exonic identity(> 0.1) and locus identity(> 0.1) with lncRNA transcript in Mouse. Here we do not take into consideration cases, where transcript shows only exonic, only locus identity or be syntenic in order not to overload the figure

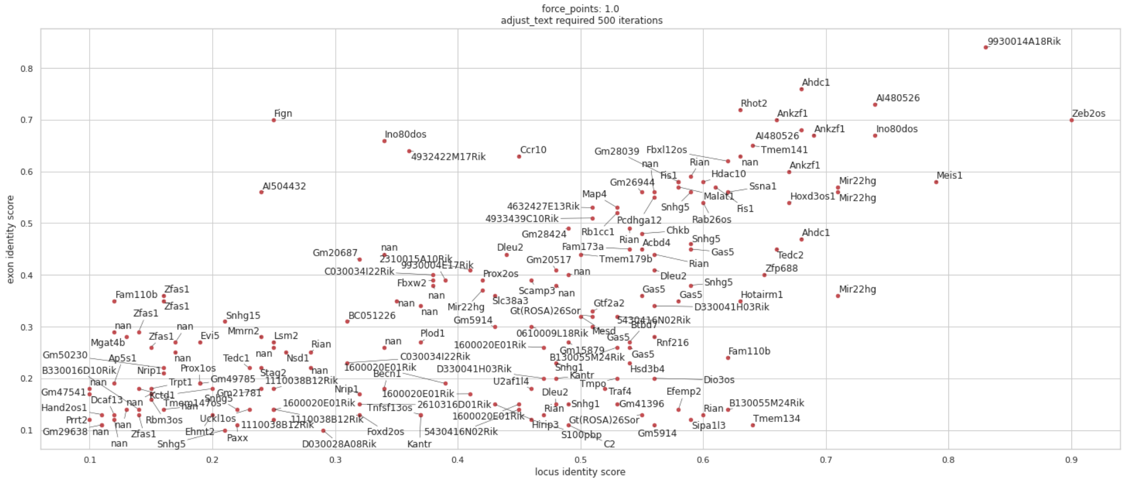

Figure 6. Mouse genes, for human transcripts, showed exonic identity(> 0.01) and locus identity(> 0.01) with lncRNA transcript in Mouse. Here we do not take into consideration cases, where transcript shows only exonic, only locus identity or be syntenic in order not to overload the figure

```
<Figure size 2160x1080 with 0 Axes>
```

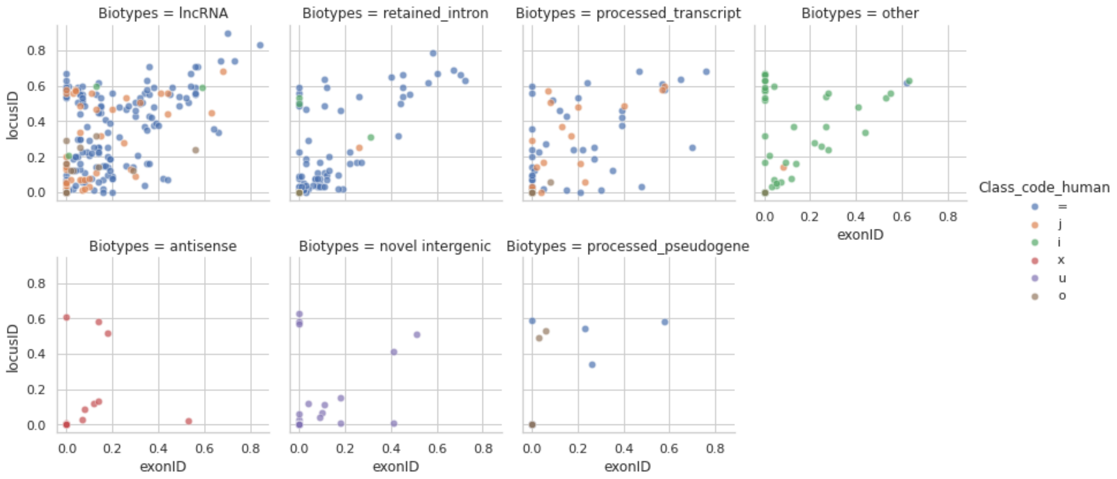

Figure 7. Locus versus exonic identities for human transcripts, divided by biotypes. Classcodes showed with color

`<Figure size 2160x1080 with 0 Axes>`

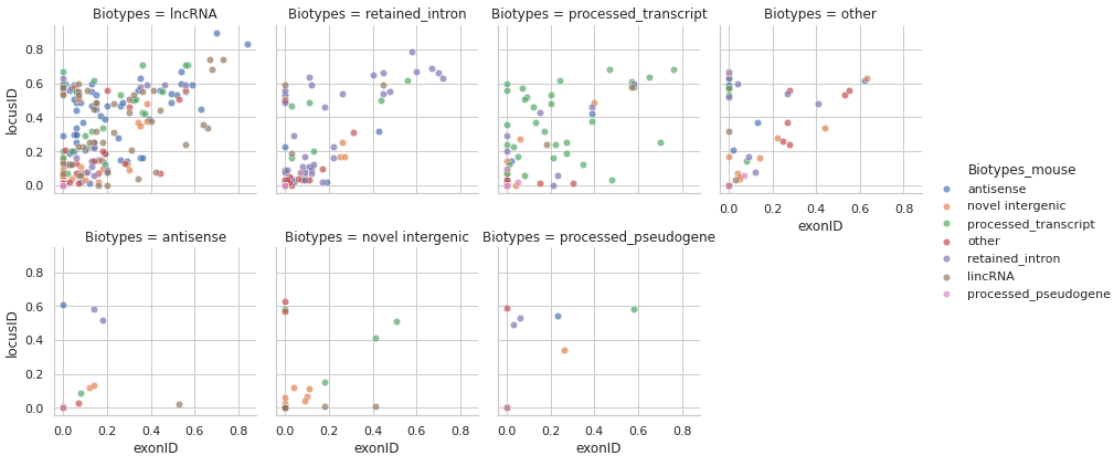

Figure 8. Locus versus exonic identities for human transcripts, divided by biotypes. Biotypes of mouse counterparts showed by color

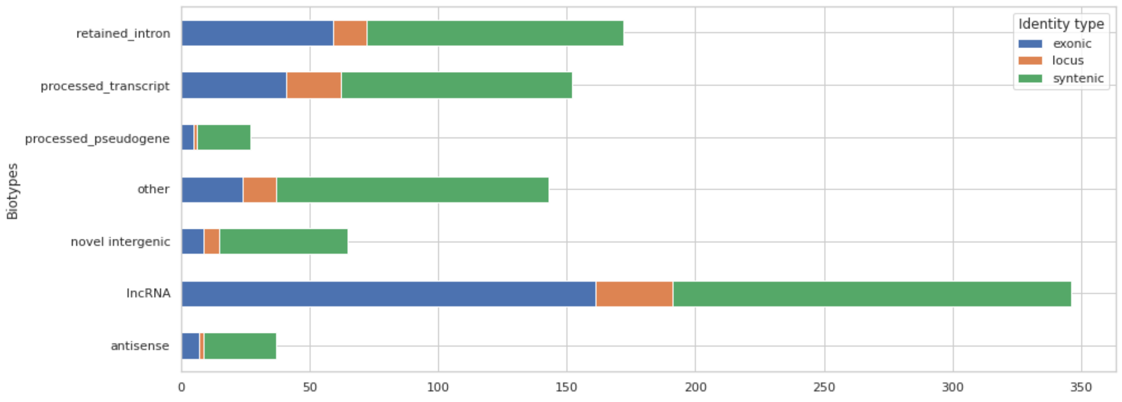

Figure 9. Biotypes of human transcripts, with the types of detected identities, e.g. if transcript showed only positional conservation - the identity is **syntenic**, if the identity on the gene level(transcript-gene alignments) only, we classify it like **locus** identity, in case when transcript showed transcript-transcript identity we classify it like **exonic**. Note, that we consider transcript **exonic** regardless it's locus identity, usually it means that the transcript with exonic identity also shows locus identity

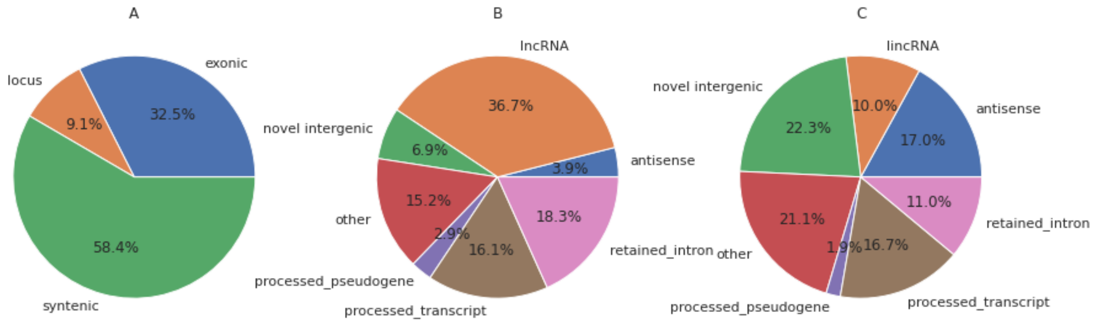

Figure 10. **A** Type of conservation. **B** Biotypes of human transcripts with found orthologs. **C** Biotypes of mouse counterpart transcripts.

2.1.1 Transcripts with highest identity

[24]:

	exonID	locusID	alignScore
count	942.000000	942.000000	9.420000e+02
mean	0.074607	0.128928	1.653443e+05
std	0.153315	0.213812	2.411879e+05
min	0.000000	0.000000	1.272700e+04
25%	0.000000	0.000000	3.603125e+04
50%	0.000000	0.000000	7.940800e+04
75%	0.070000	0.170000	1.867052e+05
max	0.840000	0.900000	2.479231e+06

2.1.2 Transcript-Transcript identity (exonID)

Transcript-Transcript identity. Across 942 transcripts, we observe 306 with detectable exonic identity, from them 39 of transcripts with the identity more than 50% of the transcript

[26]:

	Mouse	Human	exonID	Gene_Name_human	Gene_Name_mouse
484	MSTRG.44067.1	MSTRG.46437.1	0.84	PCAT1	9930014A18Rik
857	MSTRG.88532.2	MSTRG.675.5	0.76	AHDC1	Ahdc1
914	MSTRG.96012.9	MSTRG.11818.5	0.73	LINC01089	AI480526
580	MSTRG.52758.2	MSTRG.16863.5	0.72	RHOT2	Rhot2
435	MSTRG.3538.2	MSTRG.27010.3	0.70	ANKZF1	Ankzf1
742	MSTRG.67585.3	MSTRG.25929.2	0.70	FIGN	Fign
736	MSTRG.66634.1	MSTRG.25712.1	0.70	ZEB2-AS1	Zeb2os
913	MSTRG.96012.9	MSTRG.11818.3	0.68	LINC01089	AI480526
382	MSTRG.2800.1	MSTRG.26746.2	0.67	AC007383.2	Ino80dos
437	MSTRG.3538.2	MSTRG.27010.6	0.67	ANKZF1	Ankzf1
383	MSTRG.2800.2	MSTRG.26746.1	0.66	AC007383.2	Ino80dos
714	MSTRG.65545.2	MSTRG.49070.2	0.65	TMEM141	Tmem141
915	MSTRG.96013.1	MSTRG.11819.1	0.64	AC084018.1	4932422M17Rik
355	MSTRG.23001.2	MSTRG.19489.1	0.63	AC100793.2	Ccr10
424	MSTRG.33045.1	MSTRG.38706.1	0.63	ZNF346	NaN

Table 2a. Top 15 transcripts with higher transcript-transcript(exonic) identity with gene names

[27]:

	Mouse	Human	exonID	Biotypes	Biotypes_mouse
484	MSTRG.44067.1	MSTRG.46437.1	0.84	lncRNA	antisense
857	MSTRG.88532.2	MSTRG.675.5	0.76	processed_transcript	processed_transcript
914	MSTRG.96012.9	MSTRG.11818.5	0.73	lncRNA	lincRNA
580	MSTRG.52758.2	MSTRG.16863.5	0.72	retained_intron	retained_intron
435	MSTRG.3538.2	MSTRG.27010.3	0.70	retained_intron	retained_intron
742	MSTRG.67585.3	MSTRG.25929.2	0.70	processed_transcript	processed_transcript
736	MSTRG.66634.1	MSTRG.25712.1	0.70	lncRNA	antisense
913	MSTRG.96012.9	MSTRG.11818.3	0.68	lncRNA	lincRNA
382	MSTRG.2800.1	MSTRG.26746.2	0.67	lncRNA	lincRNA
437	MSTRG.3538.2	MSTRG.27010.6	0.67	retained_intron	retained_intron
383	MSTRG.2800.2	MSTRG.26746.1	0.66	lncRNA	lincRNA
714	MSTRG.65545.2	MSTRG.49070.2	0.65	processed_transcript	processed_transcript
915	MSTRG.96013.1	MSTRG.11819.1	0.64	lncRNA	lincRNA
355	MSTRG.23001.2	MSTRG.19489.1	0.63	lncRNA	antisense
424	MSTRG.33045.1	MSTRG.38706.1	0.63	other	novel intergenic

Table 2b. Top 15 transcripts with higher transcript-transcript(exonic) identity with transcript type

2.1.3 Transcript-Locus identity (locusID)

Transcript-Locus identity. Across 942 transcripts, we observe 387 with detectable exonic identity, from them 123 of transcripts with the identity more

than 50%.

[29]:

	Mouse	Human	locusID	Gene_Name_human	Gene_Name_mouse
736	MSTRG.66634.1	MSTRG.25712.1	0.90	ZEB2-AS1	Zeb2os
484	MSTRG.44067.1	MSTRG.46437.1	0.83	PCAT1	9930014A18Rik
290	MSTRG.17816.8	MSTRG.24584.3	0.79	MEIS1	Meis1
914	MSTRG.96012.9	MSTRG.11818.5	0.74	LINC01089	AI480526
382	MSTRG.2800.1	MSTRG.26746.2	0.74	AC007383.2	Ino80dos
326	MSTRG.21437.1	MSTRG.18476.1	0.71	MIR22HG	Mir22hg
325	MSTRG.21437.1	MSTRG.18476.3	0.71	MIR22HG	Mir22hg
324	MSTRG.21437.1	MSTRG.18476.6	0.71	MIR22HG	Mir22hg
437	MSTRG.3538.2	MSTRG.27010.6	0.69	ANKZF1	Ankzf1
913	MSTRG.96012.9	MSTRG.11818.3	0.68	LINC01089	AI480526
857	MSTRG.88532.2	MSTRG.675.5	0.68	AHDC1	Ahdc1
856	MSTRG.88532.2	MSTRG.675.6	0.68	AHDC1	Ahdc1
886	MSTRG.91181.6	MSTRG.44571.1	0.67	AC021097.1	Chpf2
753	MSTRG.68139.1	MSTRG.26149.1	0.67	HOXD-AS2	Hoxd3os1
436	MSTRG.3538.2	MSTRG.27010.4	0.67	ANKZF1	Ankzf1

Table 3a. Top 15 transcripts with higher transcript-genome(locus) identity

[30]:

	Mouse	Human	locusID	Biotypes	Biotypes_mouse
736	MSTRG.66634.1	MSTRG.25712.1	0.90	lncRNA	antisense
484	MSTRG.44067.1	MSTRG.46437.1	0.83	lncRNA	antisense
290	MSTRG.17816.8	MSTRG.24584.3	0.79	retained_intron	retained_intron
914	MSTRG.96012.9	MSTRG.11818.5	0.74	lncRNA	lincRNA
382	MSTRG.2800.1	MSTRG.26746.2	0.74	lncRNA	lincRNA
326	MSTRG.21437.1	MSTRG.18476.1	0.71	lncRNA	processed_transcript
325	MSTRG.21437.1	MSTRG.18476.3	0.71	lncRNA	processed_transcript
324	MSTRG.21437.1	MSTRG.18476.6	0.71	lncRNA	processed_transcript
437	MSTRG.3538.2	MSTRG.27010.6	0.69	retained_intron	retained_intron
913	MSTRG.96012.9	MSTRG.11818.3	0.68	lncRNA	lincRNA
857	MSTRG.88532.2	MSTRG.675.5	0.68	processed_transcript	processed_transcript
856	MSTRG.88532.2	MSTRG.675.6	0.68	processed_transcript	processed_transcript
886	MSTRG.91181.6	MSTRG.44571.1	0.67	lncRNA	processed_transcript
753	MSTRG.68139.1	MSTRG.26149.1	0.67	lncRNA	antisense
436	MSTRG.3538.2	MSTRG.27010.4	0.67	retained_intron	retained_intron

Table 3b. Top 15 transcripts with higher transcript-genome(locus) identity

2.2 Human transcripts conserved in Mouse (medium distance, high sensitivity, computationally expensive)

```
Shape of top final conserved human lncRNAs in Mouse  (4965, 15)
```

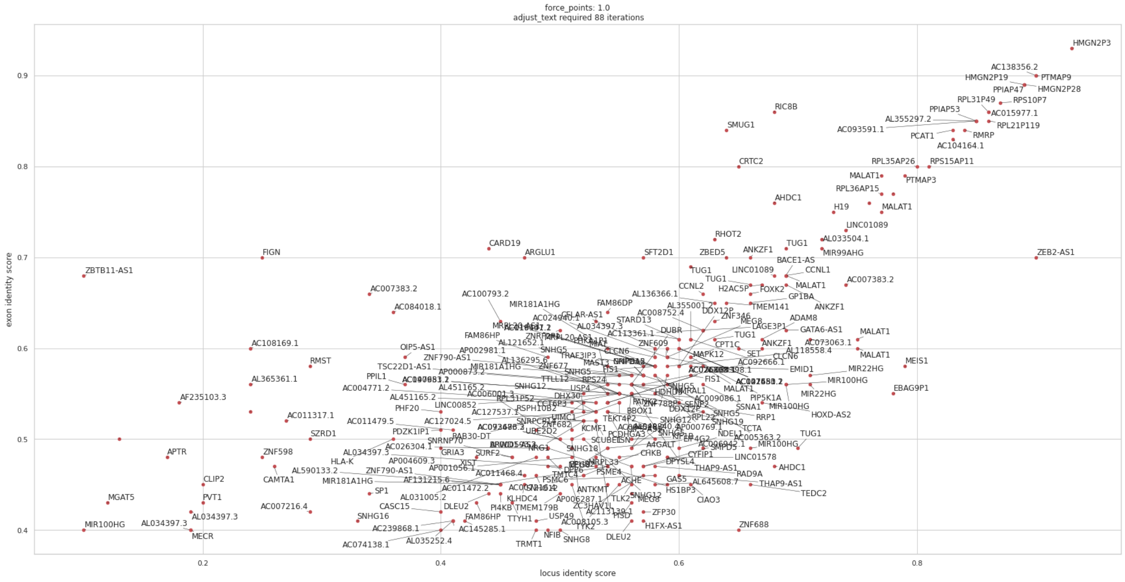

Figure 11A. Human genes, with transcripts showed exonic identity(>= 0.4) and locus identity(> 0.1) with lncRNA transcript in Mouse. Here we do not take into consideration cases, where transcript shows only exonic, only locus identity or be syntenic in order not to overload the figure

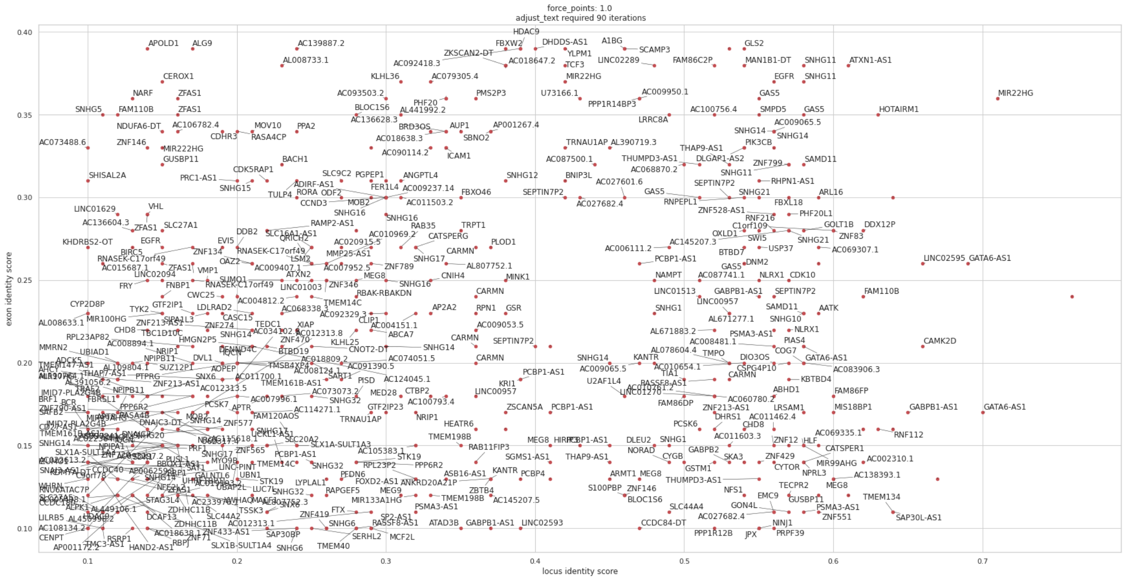

Figure 11B Human genes, with transcripts showed exonic identity(< 0.4 but > 0.1) and locus identity(> 0.1) with lncRNA transcript in Mouse. Here we do not take into consideration cases, where transcript shows only exonic, only locus identity or be syntenic in order not to overload the figure

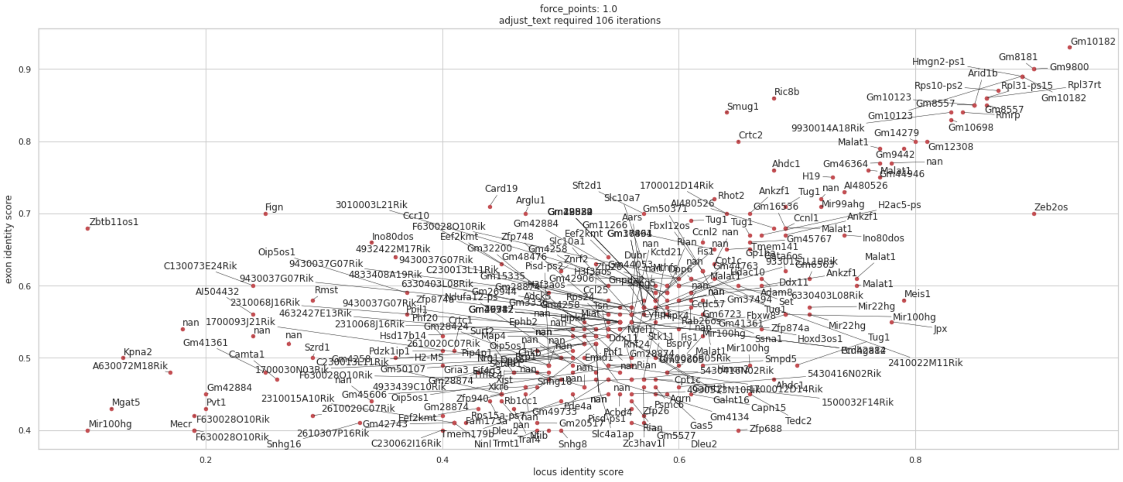

Figure 12A. Mouse genes, for human transcripts, showed exonic identity(> 0.4) and locus identity(> 0.1) with lncRNA transcript in Mouse. Here we do not take into consideration cases, where transcript shows only exonic, only locus identity or be syntenic in order not to overload the figure

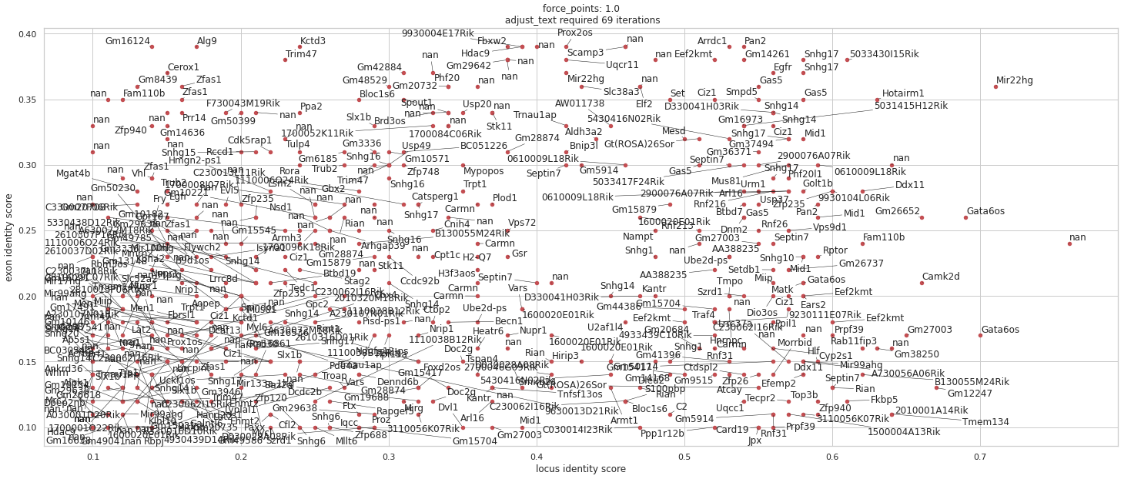

Figure 12B. Mouse genes, for human transcripts, showed exonic identity(< 0.4 but > 0.1) and locus identity(> 0.1) with lncRNA transcript in Mouse. Here we do not take into consideration cases, where transcript shows only exonic, only locus identity or be syntenic in order not to overload the figure

`<Figure size 2160x1080 with 0 Axes>`

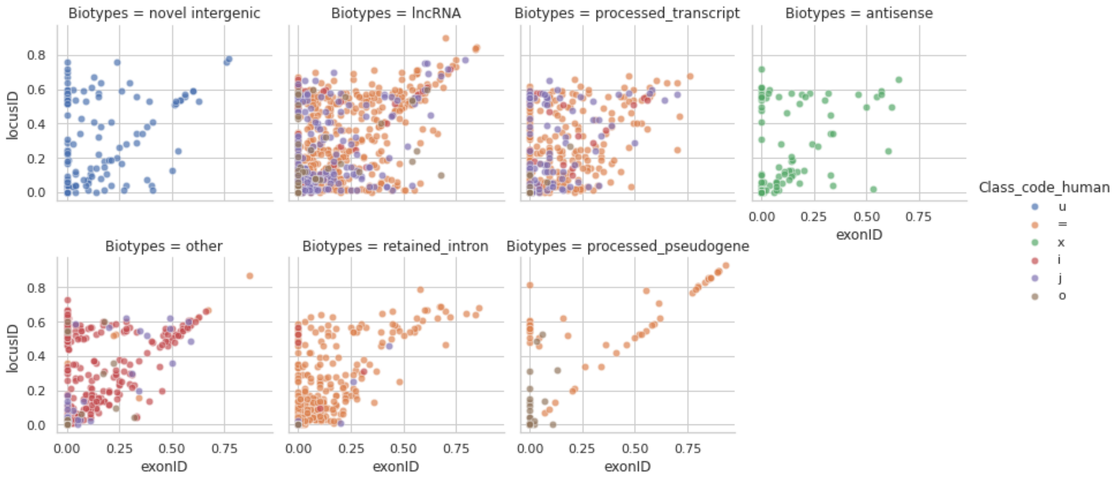

Figure 13. Locus versus exonic identities for human transcripts, divided by biotypes. Classcodes showed with color

`<Figure size 2160x1080 with 0 Axes>`

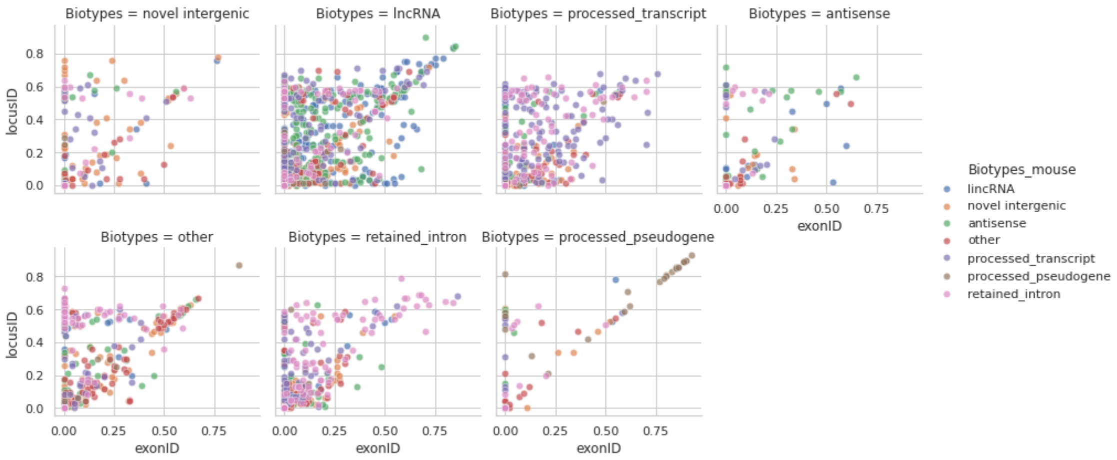

Figure 14. Locus versus exonic identities for human transcripts, divided by biotypes. Biotypes of mouse counterparts showed by color

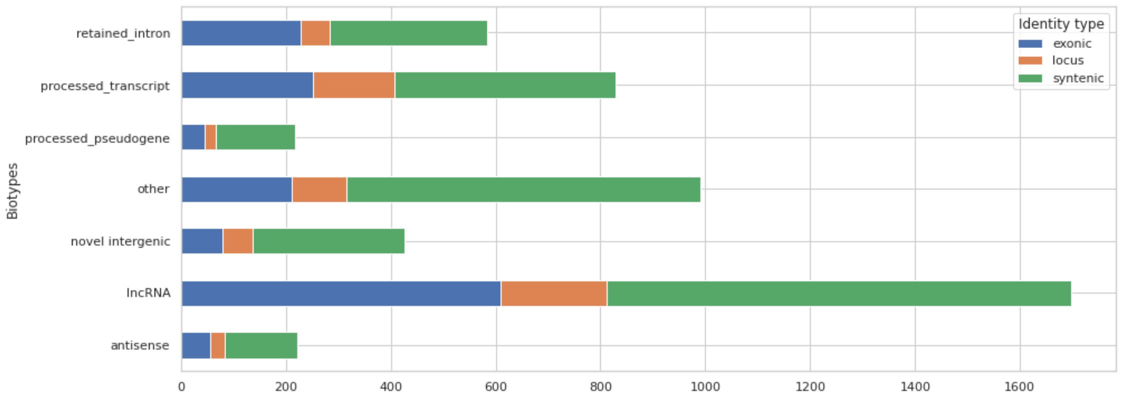

Figure 15. Biotypes of human transcripts, with the types of detected identities, e.g. if transcript showed only positional conservation - the identity is **syntenic**, if the identity on the gene level(transcript-gene alignments) only, we classify it like **locus** identity, in case when transcript showed transcript-transcript identity we classify it like **exonic**. Note, that we consider transcript **exonic** regardless it's locus identity, usually it means that the transcript with exonic identity also shows locus identity

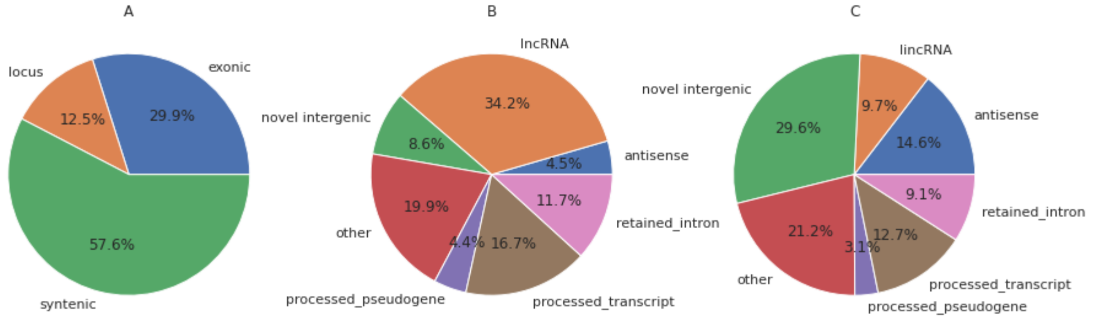

Figure 16. **A** Type of conservation. **B** Biotypes of human transcripts with found orthologs. **C** Biotypes of mouse counterpart transcripts.

2.2.1 Transcripts with highest identity

[40]:

	exonID	locusID	alignScore
count	4965.000000	4965.000000	4.965000e+03
mean	0.069498	0.123204	1.111281e+05
std	0.152731	0.209602	1.570725e+05
min	0.000000	0.000000	9.756000e+03
25%	0.000000	0.000000	3.100700e+04
50%	0.000000	0.000000	5.368600e+04
75%	0.050000	0.150000	1.404120e+05
max	0.930000	0.930000	2.479231e+06

2.2.2 Transcript-Transcript identity (exonID)

Transcript-Transcript identity. Across 4965 transcripts, we observe 1484 with detectable exonic identity, from them 201 of transcripts with the identity more than 50% of the transcript

[42]:

	Mouse	Human	exonID	Gene_Name_human	Gene_Name_mouse
875	MSTRG.120616.1	MSTRG.17420.1	0.93	HMGN2P3	Gm10182
3153	MSTRG.58882.1	MSTRG.45330.1	0.90	AC138356.2	Gm8181
2156	MSTRG.36390.1	MSTRG.9728.1	0.90	PTMAP9	Gm9800
4587	MSTRG.93846.1	MSTRG.4284.1	0.89	HMGN2P19	Hmgn2-ps1
879	MSTRG.120616.1	MSTRG.38917.1	0.89	HMGN2P28	Gm10182
1043	MSTRG.124896.1	MSTRG.16270.1	0.89	PPIAP47	Gm10123
2693	MSTRG.47969.1	MSTRG.3722.1	0.87	RPS10P7	Rps10-ps2
4667	MSTRG.95541.2	MSTRG.23888.1	0.86	AC015977.1	Rpl37rt
1320	MSTRG.14475.6	MSTRG.11539.3	0.86	RIC8B	Ric8b
3867	MSTRG.75978.1	MSTRG.11630.1	0.86	RPL31P49	Rpl31-ps15
1909	MSTRG.28957.1	MSTRG.17097.1	0.85	RPL21P119	Gm8557
1910	MSTRG.28957.1	MSTRG.35446.1	0.85	AC093591.1	Gm8557
1042	MSTRG.124896.1	MSTRG.18863.1	0.85	PPIAP53	Gm10123
2790	MSTRG.51489.1	MSTRG.41582.1	0.85	AL355297.2	Arid1b
2613	MSTRG.46764.2	MSTRG.10634.4	0.84	SMUG1	Smug1

Table 4a. Top 15 transcripts with higher transcript-transcript(exonic) identity with gene names

[43]:

	Mouse	Human	exonID	Biotypes	Biotypes_mouse
875	MSTRG.120616.1	MSTRG.17420.1	0.93	processed_pseudogene	processed_pseudogene
3153	MSTRG.58882.1	MSTRG.45330.1	0.90	processed_pseudogene	processed_pseudogene
2156	MSTRG.36390.1	MSTRG.9728.1	0.90	processed_pseudogene	processed_pseudogene
4587	MSTRG.93846.1	MSTRG.4284.1	0.89	processed_pseudogene	processed_pseudogene
879	MSTRG.120616.1	MSTRG.38917.1	0.89	processed_pseudogene	processed_pseudogene
1043	MSTRG.124896.1	MSTRG.16270.1	0.89	processed_pseudogene	processed_pseudogene
2693	MSTRG.47969.1	MSTRG.3722.1	0.87	other	processed_pseudogene
4667	MSTRG.95541.2	MSTRG.23888.1	0.86	processed_pseudogene	processed_transcript
1320	MSTRG.14475.6	MSTRG.11539.3	0.86	retained_intron	processed_transcript
3867	MSTRG.75978.1	MSTRG.11630.1	0.86	processed_pseudogene	processed_pseudogene
1909	MSTRG.28957.1	MSTRG.17097.1	0.85	processed_pseudogene	processed_pseudogene
1910	MSTRG.28957.1	MSTRG.35446.1	0.85	processed_pseudogene	processed_pseudogene
1042	MSTRG.124896.1	MSTRG.18863.1	0.85	processed_pseudogene	processed_pseudogene
2790	MSTRG.51489.1	MSTRG.41582.1	0.85	lncRNA	antisense
2613	MSTRG.46764.2	MSTRG.10634.4	0.84	retained_intron	retained_intron

Table 4b. Top 15 transcripts with higher transcript-transcript(exonic) identity with transcript type

2.2.3 Transcript-Locus identity (locusID)

Transcript-Locus identity. Across 4965 transcripts, we observe 2078 with detectable exonic identity, from them 643 of transcripts with the identity more

than 50%.

[45]:

	Mouse	Human	locusID	Gene_Name_human	Gene_Name_mouse
875	MSTRG.120616.1	MSTRG.17420.1	0.93	HMGN2P3	Gm10182
3524	MSTRG.66634.1	MSTRG.25712.1	0.90	ZEB2-AS1	Zeb2os
3153	MSTRG.58882.1	MSTRG.45330.1	0.90	AC138356.2	Gm8181
2156	MSTRG.36390.1	MSTRG.9728.1	0.90	PTMAP9	Gm9800
4587	MSTRG.93846.1	MSTRG.4284.1	0.89	HMGN2P19	Hmgn2-ps1
879	MSTRG.120616.1	MSTRG.38917.1	0.89	HMGN2P28	Gm10182
1043	MSTRG.124896.1	MSTRG.16270.1	0.89	PPIAP47	Gm10123
2693	MSTRG.47969.1	MSTRG.3722.1	0.87	RPS10P7	Rps10-ps2
4667	MSTRG.95541.2	MSTRG.23888.1	0.86	AC015977.1	Rpl37rt
1909	MSTRG.28957.1	MSTRG.17097.1	0.86	RPL21P119	Gm8557
3867	MSTRG.75978.1	MSTRG.11630.1	0.86	RPL31P49	Rpl31-ps15
2790	MSTRG.51489.1	MSTRG.41582.1	0.85	AL355297.2	Arid1b
1910	MSTRG.28957.1	MSTRG.35446.1	0.85	AC093591.1	Gm8557
1042	MSTRG.124896.1	MSTRG.18863.1	0.85	PPIAP53	Gm10123
4140	MSTRG.84185.1	MSTRG.47509.1	0.84	RMRP	Rmrp

Table 5a Top 15 transcripts with higher transcript-genome(locus) identity

[46]:

	Mouse	Human	locusID	Biotypes	Biotypes_mouse
875	MSTRG.120616.1	MSTRG.17420.1	0.93	processed_pseudogene	processed_pseudogene
3524	MSTRG.66634.1	MSTRG.25712.1	0.90	lncRNA	antisense
3153	MSTRG.58882.1	MSTRG.45330.1	0.90	processed_pseudogene	processed_pseudogene
2156	MSTRG.36390.1	MSTRG.9728.1	0.90	processed_pseudogene	processed_pseudogene
4587	MSTRG.93846.1	MSTRG.4284.1	0.89	processed_pseudogene	processed_pseudogene
879	MSTRG.120616.1	MSTRG.38917.1	0.89	processed_pseudogene	processed_pseudogene
1043	MSTRG.124896.1	MSTRG.16270.1	0.89	processed_pseudogene	processed_pseudogene
2693	MSTRG.47969.1	MSTRG.3722.1	0.87	other	processed_pseudogene
4667	MSTRG.95541.2	MSTRG.23888.1	0.86	processed_pseudogene	processed_transcript
1909	MSTRG.28957.1	MSTRG.17097.1	0.86	processed_pseudogene	processed_pseudogene
3867	MSTRG.75978.1	MSTRG.11630.1	0.86	processed_pseudogene	processed_pseudogene
2790	MSTRG.51489.1	MSTRG.41582.1	0.85	lncRNA	antisense
1910	MSTRG.28957.1	MSTRG.35446.1	0.85	processed_pseudogene	processed_pseudogene
1042	MSTRG.124896.1	MSTRG.18863.1	0.85	processed_pseudogene	processed_pseudogene
4140	MSTRG.84185.1	MSTRG.47509.1	0.84	lncRNA	lincRNA

Table 5b Top 15 transcripts with higher transcript-genome(locus) identity, biotypes

2.3 Mouse transcripts conserved in Human

```
Shape of top final conserved human lncRNAs in Mouse  (1012, 15)
```

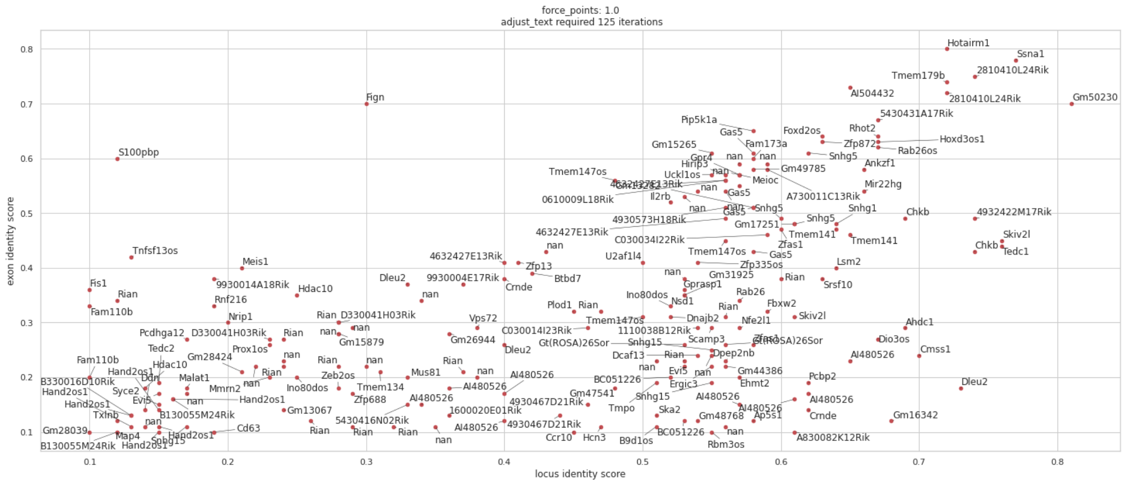

Figure 17. Mouse genes, with transcripts showed exonic identity(> 0.1) and locus identity(> 0.1) with lncRNA transcript in Human. Here we do not take into consideration cases, where transcript shows only exonic, only locus identity or be syntenic in order not to overload the figure

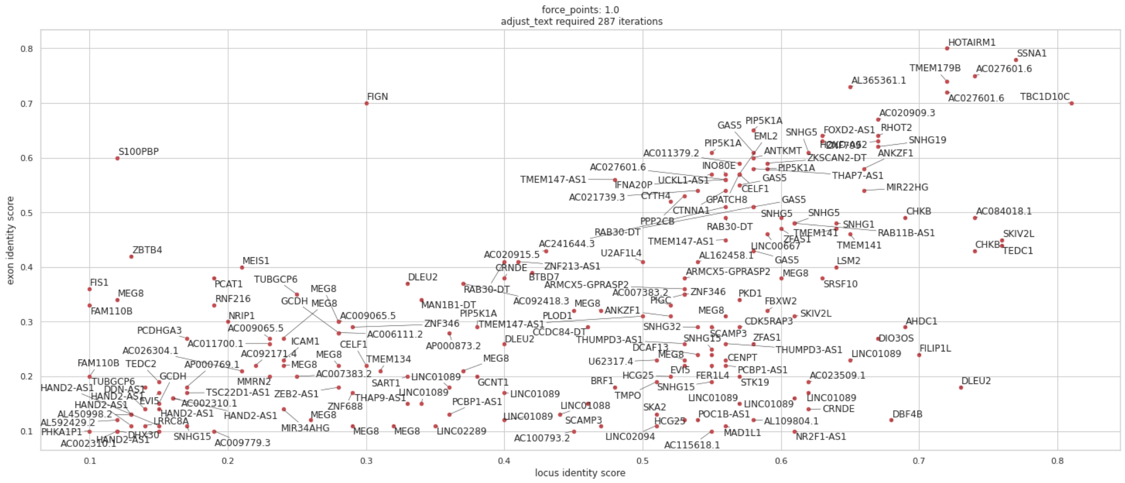

Figure 18. Mouse genes, for human transcripts, showed exonic identity(> 0.01) and locus identity(> 0.01) with lncRNA transcript in Mouse. Here we do not take into consideration cases, where transcript shows only exonic, only locus identity or be syntenic in order not to overload the figure

`<Figure size 2160x1080 with 0 Axes>`

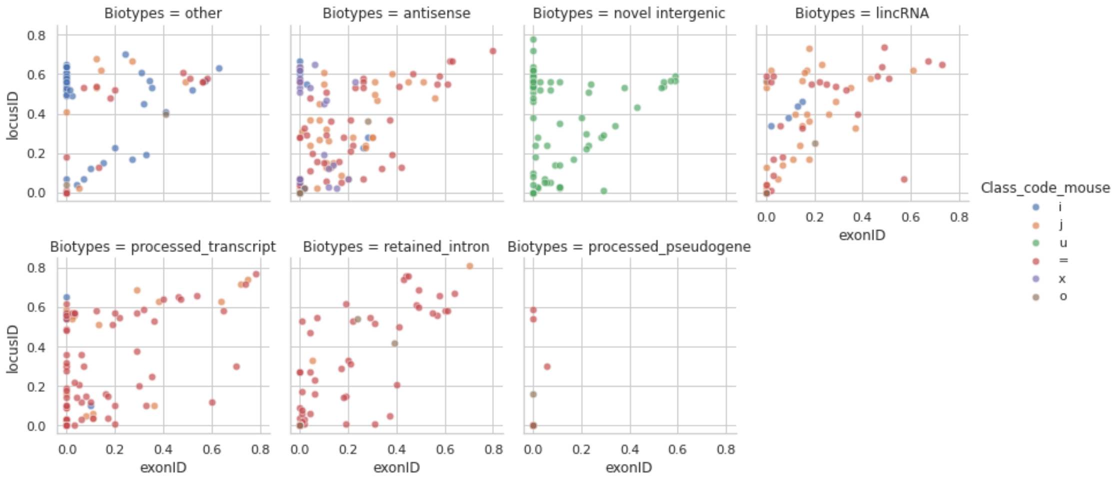

Figure 19. Locus versus exonic identities for mouse transcripts, divided by biotypes. Classcodes showed with color

<Figure size 2160x1080 with 0 Axes>

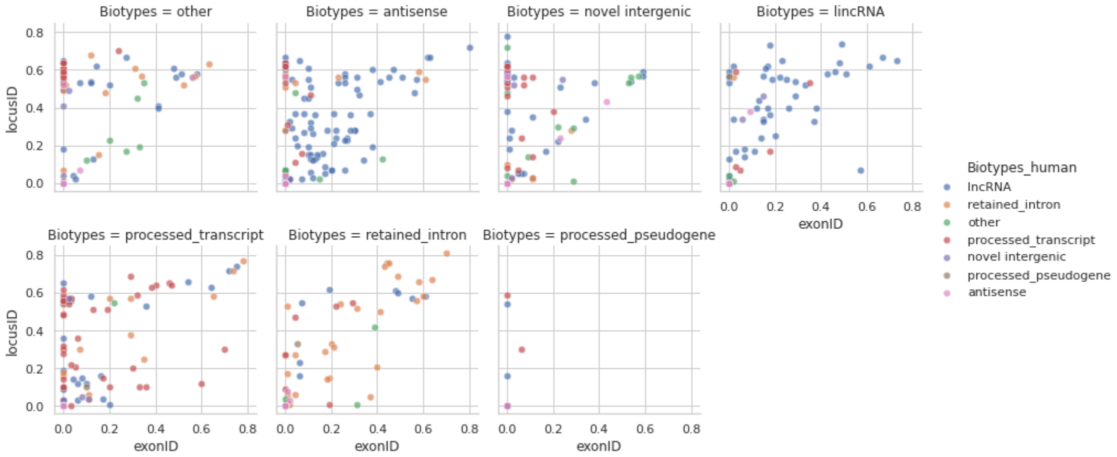

Figure 20. Locus versus exonic identities for mouse transcripts, divided by biotypes. Biotypes of human counterparts showed by color

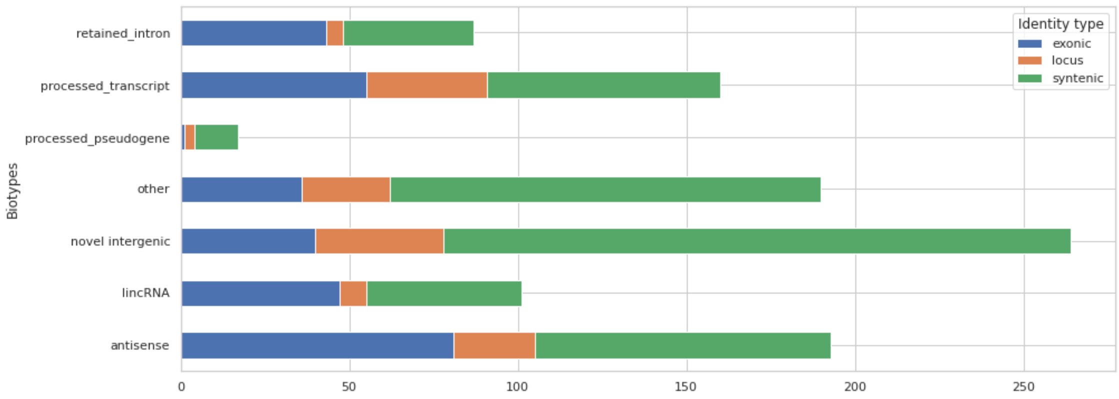

Figure 21. Biotypes of human transcripts, with the types of detected identities, e.g. if transcript showed only positional conservation - the identity is **syntenic**, if the identity on the gene level(transcript-gene alignments) only, we classify it like **locus** identity, in case when transcript showed transcript-transcript identity we classify it like **exonic**. Note, that we consider transcript **exonic** regardless it's locus identity, usually it means that the transcript with exonic identity also shows locus identity

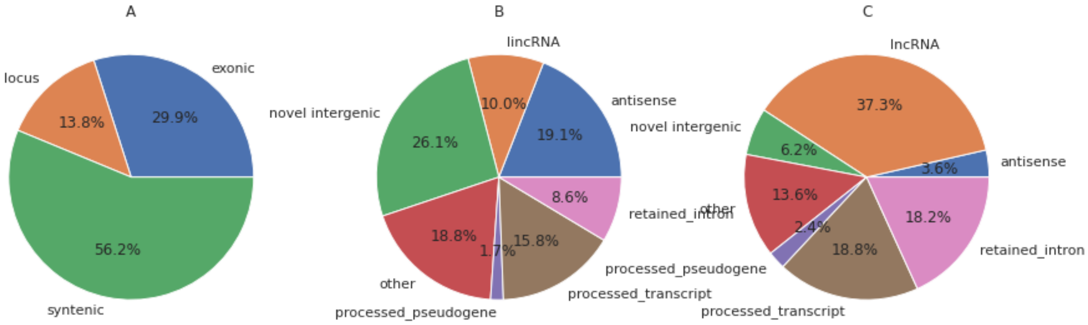

Figure 22. A Type of conservation. **B** Biotypes of mouse transcripts with found orthologs. **C** Biotypes of human counterpart transcripts.

2.3.1 Transcripts with highest identity

2.3.2 Transcript-Transcript identity (exonID)

```
Transcript-Transcript identity. Across 1012 transcripts, we observe 303 with
detectable exonic identity, from them 43 of transcripts with the identity more
than 50% of the transcript
```

[56]:

	Mouse	Human	exonID	Gene_Name_human	Gene_Name_mouse
753	MSTRG.100138.1	MSTRG.42449.1	0.80	HOTAIRM1	Hotairm1
866	MSTRG.65491.2	MSTRG.49120.1	0.78	SSNA1	Ssna1
297	MSTRG.24323.2	MSTRG.20387.1	0.75	AC027601.6	2810410L24Rik
936	MSTRG.61669.1	MSTRG.7766.2	0.74	TMEM179B	Tmem179b
362	MSTRG.79670.1	MSTRG.2244.1	0.73	AL365361.1	AI504432
298	MSTRG.24323.3	MSTRG.20387.1	0.72	AC027601.6	2810410L24Rik
438	MSTRG.67585.3	MSTRG.25929.2	0.70	FIGN	Fign
965	MSTRG.61224.1	MSTRG.7977.4	0.70	TBC1D10C	Gm50230
392	MSTRG.107429.1	MSTRG.23049.1	0.67	AC020909.3	5430431A17Rik
478	MSTRG.78756.4	MSTRG.2759.5	0.65	PIP5K1A	Pip5k1a
46	MSTRG.87376.3	MSTRG.1227.1	0.64	FOXD2-AS1	Foxd2os
158	MSTRG.52758.2	MSTRG.16863.5	0.64	RHOT2	Rhot2
448	MSTRG.68139.1	MSTRG.26149.1	0.63	HOXD-AS2	Hoxd3os1
330	MSTRG.120606.1	MSTRG.21818.1	0.63	ZNF799	Zfp872
164	MSTRG.52642.1	MSTRG.16941.1	0.62	SNHG19	Rab26os

Table 6a. Top 15 transcripts with highest transcript-transcript(exonic) identity with gene names

[57]:

	Mouse	Human	exonID	Biotypes	Biotypes_human
753	MSTRG.100138.1	MSTRG.42449.1	0.80	antisense	lncRNA
866	MSTRG.65491.2	MSTRG.49120.1	0.78	processed_transcript	retained_intron
297	MSTRG.24323.2	MSTRG.20387.1	0.75	processed_transcript	lncRNA
936	MSTRG.61669.1	MSTRG.7766.2	0.74	processed_transcript	retained_intron
362	MSTRG.79670.1	MSTRG.2244.1	0.73	lincRNA	lncRNA
298	MSTRG.24323.3	MSTRG.20387.1	0.72	processed_transcript	lncRNA
438	MSTRG.67585.3	MSTRG.25929.2	0.70	processed_transcript	processed_transcript
965	MSTRG.61224.1	MSTRG.7977.4	0.70	retained_intron	retained_intron
392	MSTRG.107429.1	MSTRG.23049.1	0.67	lincRNA	lncRNA
478	MSTRG.78756.4	MSTRG.2759.5	0.65	processed_transcript	retained_intron
46	MSTRG.87376.3	MSTRG.1227.1	0.64	processed_transcript	lncRNA
158	MSTRG.52758.2	MSTRG.16863.5	0.64	retained_intron	retained_intron
448	MSTRG.68139.1	MSTRG.26149.1	0.63	antisense	lncRNA
330	MSTRG.120606.1	MSTRG.21818.1	0.63	other	retained_intron
164	MSTRG.52642.1	MSTRG.16941.1	0.62	antisense	lncRNA

Table 6b Top 15 transcripts with highest transcript-transcript(exonic) identity with transcript type

2.3.3 Transcript-Locus identity (locusID)

[58]:

	Mouse	Human	locusID	Gene_Name_human	Gene_Name_mouse
965	MSTRG.61224.1	MSTRG.7977.4	0.81	TBC1D10C	Gm50230
468	MSTRG.72288.1	MSTRG.27386.2	0.78	NRSN2-AS1	NaN
866	MSTRG.65491.2	MSTRG.49120.1	0.77	SSNA1	Ssna1
689	MSTRG.53536.3	MSTRG.39579.1	0.76	SKIV2L	Skiv2l
124	MSTRG.30163.1	MSTRG.14988.2	0.76	TEDC1	Tedc1
41	MSTRG.96013.1	MSTRG.11819.1	0.74	AC084018.1	4932422M17Rik
297	MSTRG.24323.2	MSTRG.20387.1	0.74	AC027601.6	2810410L24Rik
533	MSTRG.45872.5	MSTRG.30234.5	0.74	CHKB	Chkb
50	MSTRG.39000.7	MSTRG.12662.4	0.73	DLEU2	Dleu2
298	MSTRG.24323.3	MSTRG.20387.1	0.72	AC027601.6	2810410L24Rik
753	MSTRG.100138.1	MSTRG.42449.1	0.72	HOTAIRM1	Hotairm1
577	MSTRG.77446.1	MSTRG.32949.1	0.72	RSRC1	NaN
936	MSTRG.61669.1	MSTRG.7766.2	0.72	TMEM179B	Tmem179b
564	MSTRG.49685.1	MSTRG.31840.1	0.70	FILIP1L	Cmss1
534	MSTRG.45872.2	MSTRG.30234.5	0.69	CHKB	Chkb

Table 7a Top 15 transcripts with highest transcript-genome(locus) identity

[59]:

	Mouse	Human	locusID	Biotypes	Biotypes_human
965	MSTRG.61224.1	MSTRG.7977.4	0.81	retained_intron	retained_intron
468	MSTRG.72288.1	MSTRG.27386.2	0.78	novel intergenic	lncRNA
866	MSTRG.65491.2	MSTRG.49120.1	0.77	processed_transcript	retained_intron
689	MSTRG.53536.3	MSTRG.39579.1	0.76	retained_intron	retained_intron
124	MSTRG.30163.1	MSTRG.14988.2	0.76	retained_intron	retained_intron
41	MSTRG.96013.1	MSTRG.11819.1	0.74	lincRNA	lncRNA
297	MSTRG.24323.2	MSTRG.20387.1	0.74	processed_transcript	lncRNA
533	MSTRG.45872.5	MSTRG.30234.5	0.74	retained_intron	retained_intron
50	MSTRG.39000.7	MSTRG.12662.4	0.73	lincRNA	lncRNA
298	MSTRG.24323.3	MSTRG.20387.1	0.72	processed_transcript	lncRNA
753	MSTRG.100138.1	MSTRG.42449.1	0.72	antisense	lncRNA
577	MSTRG.77446.1	MSTRG.32949.1	0.72	novel intergenic	other
936	MSTRG.61669.1	MSTRG.7766.2	0.72	processed_transcript	retained_intron
564	MSTRG.49685.1	MSTRG.31840.1	0.70	other	processed_transcript
534	MSTRG.45872.2	MSTRG.30234.5	0.69	retained_intron	retained_intron

Table 7b Top 15 transcripts with highest transcript-genome(locus) identity, biotypes

3 Comparison of obtained datasets

3.1 Comparison of pairs human-mouse and mouse-human

3.1.1 Level of transcript pairs human-mouse and mouse-human

Here we would like to compare the dataset of human transcripts with orthologous transcripts in mouse versus the dataset of mouse transcripts with orthologs in human

[60]:

Feature	Human-Mouse	Mouse-Human	Difference
Total number of pairs	942	1012	70
Number of unique human transcripts	942	663	279
Number of unique mouse transcripts	668	1012	344
Unique human genes(without novel)	692	564	128
Unique mouse genes(without novel)	461	560	99
Novel human interginic transcripts	65	53	12
Novel mouse interginic transcripts	163	264	101
Conservation exonic, transcripts	306	303	3
Conservation locus, transcripts	387	442	55
Conservation syntenic(positional)	550	569	19

Table 8. Comparison of two datasets

Slncky uses efficient and sensitive strategy to align lncRNAs and characterize their sequence and transcript evolution. To this end, slncky identifies the syntenic genomic region for a lncRNA in the orthologous species. If a transcript exists in a syntenic region, slncky aligns the two regions using a sensitive seed-based local pairwise aligner. To avoid the possibility of spurious matches, slncky scores each alignment relative to a set of random intergenic regions from the orthologous genome and only keeps alignments that score higher than 95 % of the random intergenic sequences.

The out.orthologs.top.txt file contains only the best alignment (based on exonic identity) for each lncRNA. So in case when we have GAS5 gene in human with 6 isoforms and gene Gas5 in Mouse with 4 isoforms for analysis human-mouse we are trying to align each of 6 human transcripts to each of 4 transcripts in Mouse, and report the best results for each out of 6 human transcripts. In the case of mouse-human we have only 4 isoforms to check, so we can report only 4 pairs.

[61]:

	Human	Mouse	alignScore	exonID	locusID
797	MSTRG.3371.5	MSTRG.7623.2	290760.0	0.36	0.55
798	MSTRG.3371.4	MSTRG.7623.3	290241.0	0.35	0.58
799	MSTRG.3371.7	MSTRG.7623.3	290760.0	0.26	0.54
800	MSTRG.3371.3	MSTRG.7623.4	290241.0	0.30	0.51
801	MSTRG.3371.6	MSTRG.7623.4	290241.0	0.45	0.59
802	MSTRG.3371.1	MSTRG.7623.4	45642.0	0.07	0.23

Table 9. Transcript pairs for human transcripts with conserved counterpart in mouse

[62]:

	Mouse	Human	alignScore	exonID	locusID
582	MSTRG.7623.1	MSTRG.3371.1	95433.0	0.55	0.57
583	MSTRG.7623.3	MSTRG.3371.1	95469.0	0.61	0.58
584	MSTRG.7623.2	MSTRG.3371.1	95469.0	0.51	0.58
585	MSTRG.7623.4	MSTRG.3371.1	95469.0	0.43	0.58

Table 10. Transcript pairs for mouse transcripts with conserved counterpart in human

[136]:

	pairs
reciprocal	537
not in Human-Mouse	399
other	187
isoforms of same human gene	149
not in Mouse-Human	66
isoforms(both in antisense to reference gene)	3

Table 11. Comparison of the pairs of transcripts between two datasets

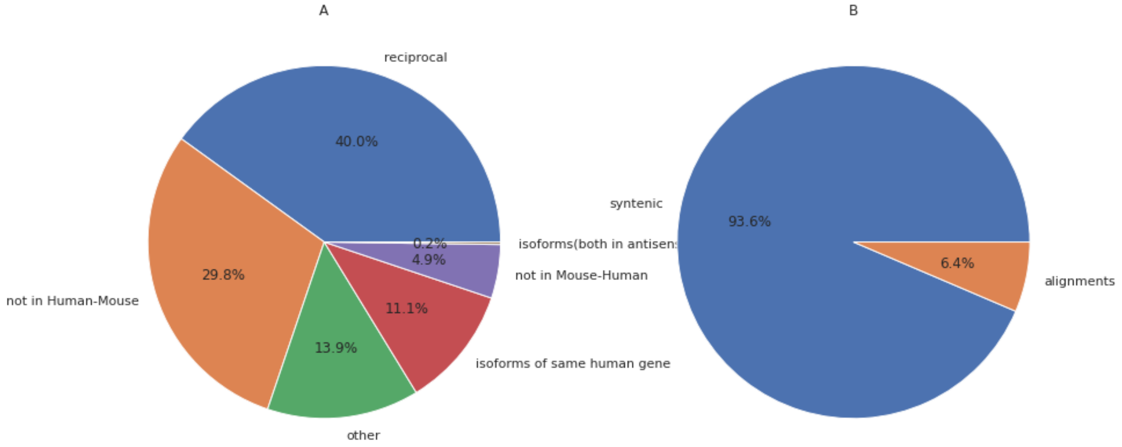

Figure 23 **A** Comparison of transcript pairs Human-Mouse and Mouse-Human **B** Distribution of transcripts from category "other", e.g cases where we have different human transcripts aligned to the same mouse transcript.

Group "reciprocal" represents the exact match between pairs of transcripts.

Group "not in Human-Mouse" represents pairs of transcripts, existed only when we are looking for mouse orthologs in human

Group "not in Mouse-Human" represents pairs of transcripts, existed only when we are looking for human orthologs in mouse

Group "isoforms of same human gene" represents pairs of transcripts, where human transcripts belongs to the same reference gene

Group "isoforms(both in antisense to reference gene)" represents pairs of transcripts, where in both cases human transcripts in antisense to reference gene

Category "other" represents the cases where we have the same mouse transcript, but different human transcripts for Human-Mouse and Mouse-Human pairs

Category "other" may be divided into two groups - syntenic transcripts and transcripts with alignments for both pairs(H-M and M-H) Now we are interested to check cases where both pairs of transcripts share the same Mouse transcript with locus and exonic alignments

[67]:

	Gene_Name_humanHM	Gene_Name_humanMH	Class_code intercept
61	AC010642.2	AC020915.5	= / x
141	HIRIP3	INO80E	= / =
143	ITGAL	AC002310.1	i / =
311	U73166.1	SEMA3B	= / =
463	CDK5RAP3	SP2-AS1	= / =
638	RBM23	AL132780.1	= / j
650	DLEU1	DLEU2	= / j
715	MAPK12	TUBGCP6	j / =
789	TSC2	PKD1	= / =
821	ANGPTL4	HCG25	i / =
853	CSNK2B	C6orf47-AS1	= / =
868	AL365205.1	USP49	= / i

Table 12. Cases where both pairs of transcripts share the same Mouse transcript with locus and exonic alignments.

All of these genes either overlap each other(for example HIRIP3 and INO80E), or located close to each other(U73166.1 and SEMA3B). More complicated case is AC010642.2 and AC020915.5, which are overlap each other on the opposite strands, and according to our prediction one of our transcripts with reference AC020915.5 have a classcode “x”, e.g. our predicted transcript is on the same strand as AC010642.2

The pair ANGPTL4 and HCG25 look strange, let’s check it

First mouse transcripts in human:

[68]:

	Human	Mouse	Gene_Name_human	Gene_Name_mouse
694	MSTRG.39587.1	MSTRG.53399.1	HCG25	BC051226

And now human transcripts in Mouse

[69]:

	Human	Mouse	Gene_Name_human	Gene_Name_mouse
588	MSTRG.21670.1	MSTRG.53399.1	ANGPTL4	BC051226
589	MSTRG.39587.1	MSTRG.53399.1	HCG25	BC051226

The source of strange behaviour is that we have one additional human transcript(of other gene) which align to the same mouse transcript.

3.1.2 Level of data-sets

Here rather to look at pairs of transcripts we compare the distributions of the data

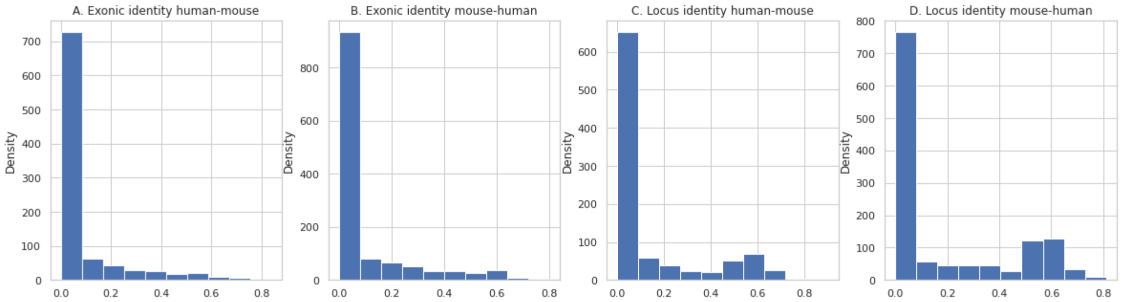

Figure 24. Comparison of the identity score distributions for human-mouse versus mouse-human datasets

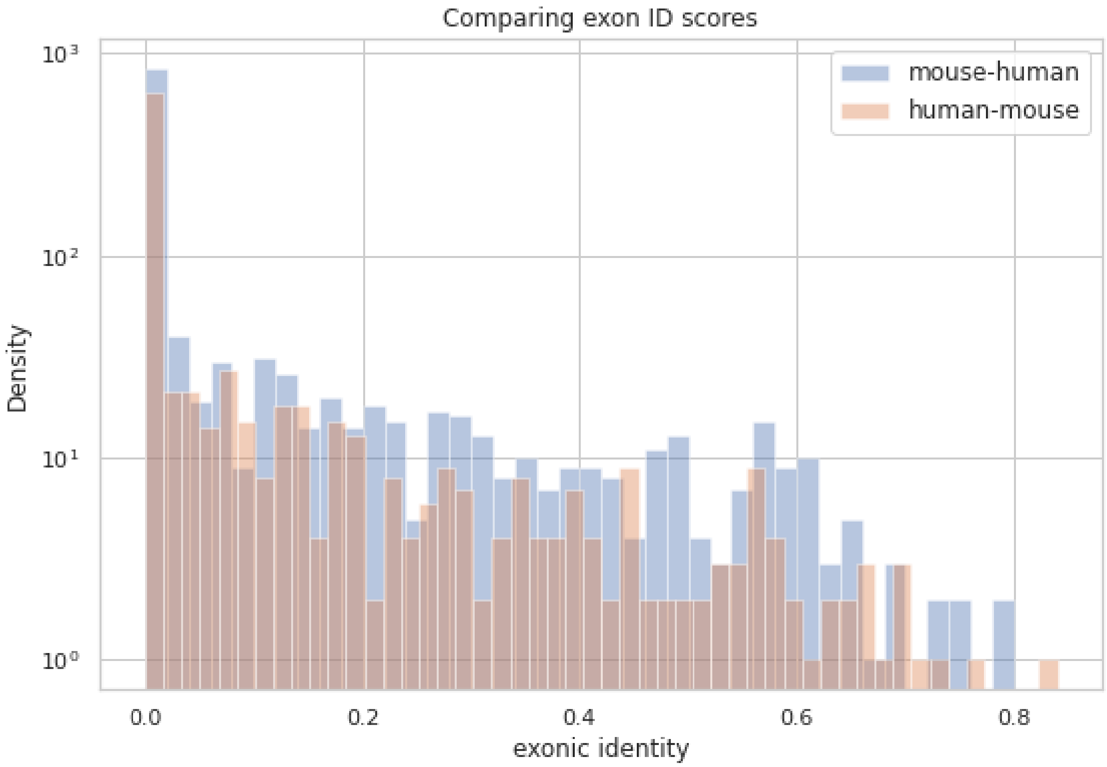

Figure 25. Comparison of the exone identity score distributions (log scale)

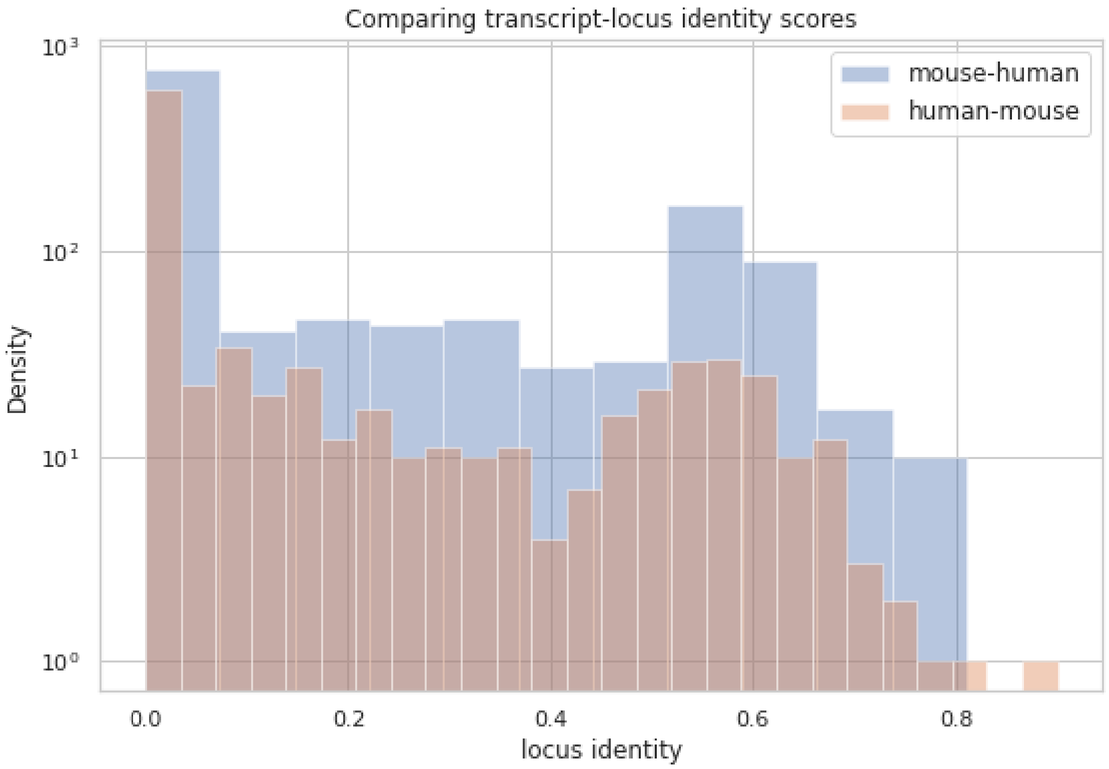

Figure 26. Comparison of the locus identity score distributions (log scale)

3.1.3 Summary

In this part we briefly describe the main difference between two datasets - human orthologs in mice and vice versa. First we check what data is common for both dataset, what are the main differences and explain them on the level of human-mouse transcript pairs, also we compare distributions of identity scores. We found that more than half of the pairs(reciprocal + other isoforms of the same gene) are the same in both datasets. Higher difference observed in group of transcripts which are not present in human-mouse dataset, due to slncky design(it looks for best counterpart for each transcript across second species noncoding transcriptome) and much larger transcriptome in Mouse(10716 vs 25223). Size of transcriptome strongly depends on the type and quality of the libraries used for assembly, as well as number of used libraries. From our previous experience from syntDB we know that using more libraries led to flatting of that difference.

3.2 Comparison of human-mouse datasets (fast vs sensitive options)

3.2.1 Genearal

[73]:

Feature	Human-Mouse_fast	Human-Mouse_sensitive	Difference
Total number of pairs	942	4965	4023
Number of unique human transcripts	942	4965	4023
Number of unique mouse transcripts	668	3200	2532
Unique human genes(without novel)	692	3529	2837
Unique mouse genes(without novel)	461	1934	1473
Novel human interginic transcripts	65	427	362
Novel mouse interginic transcripts	163	1035	872
Conservation exonic, transcripts	306	1484	1178
Conservation locus, transcripts	387	2078	1691
Conservation syntenic(positional)	550	2861	2311

Please note, that the difference between fast and sensitive methods strongly depends on the distance between species, closer species tends to show closer results, but with the growing distance the difference will grow. The disadvantage of the sensitive option is the fact that the computations are resource heavy (CPU hours vs CPU weeks). Another option is to use chain files from UCSC, hence have the results similar to sensitive options and reduce computation time to CPU days, the limitation factor - chain files available only for some species and genome versions.

3.2.2 Comparison of human transcript biotypes

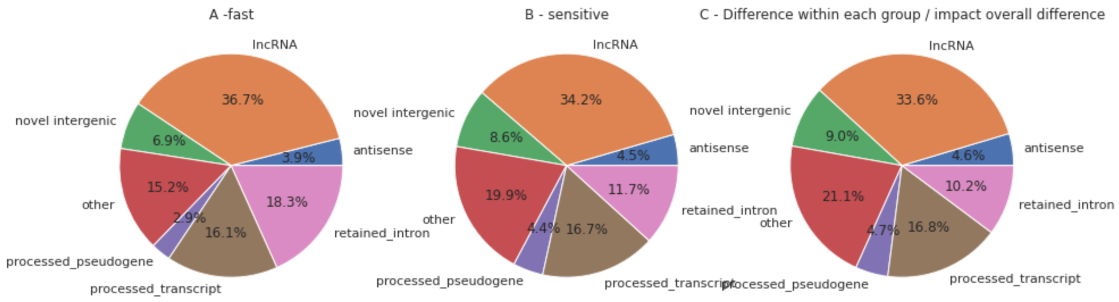

Figure 27. **A** Distribution of human transcript biotypes for fast approach **B** Distribution of human transcript biotypes for sensitive approach **C** Overall difference between both datasets based on difference within each biotype group

3.2.3 Comparison conservation properties, regarding to transcript biotype

```
<Figure size 2880x1080 with 0 Axes>
```

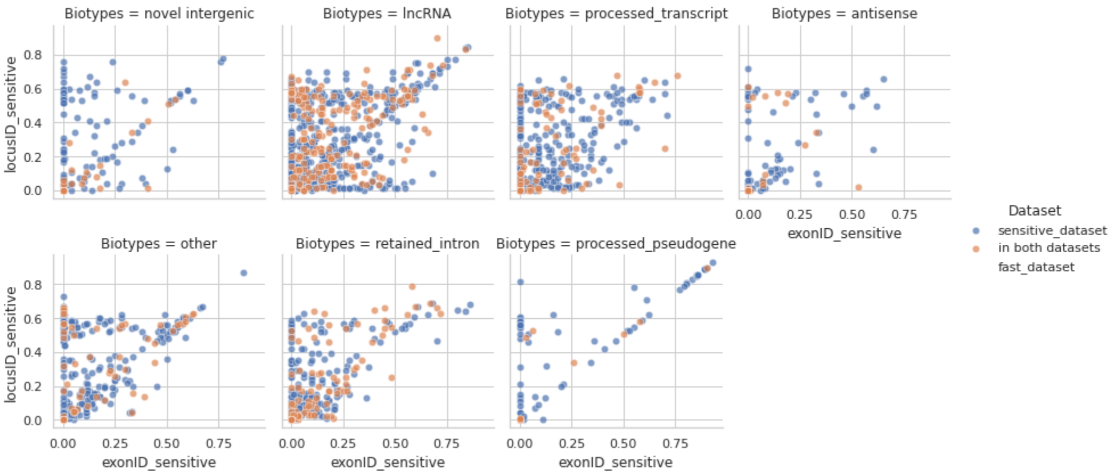

Figure 28. Mapping of exonic vs locus identity scores for sensitive datasets, divided by biotypes. Transcripts common for both datasets showed by color

`<Figure size 2880x1080 with 0 Axes>`

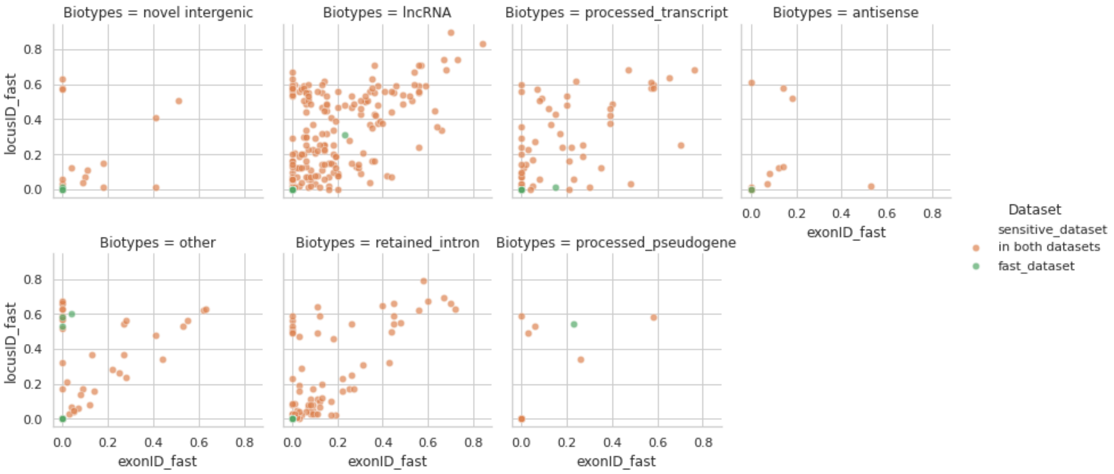

Figure 29. Mapping of exonic vs locus identity scores for fast datasets, divided by biotypes. Transcripts common for both datasets showed by color

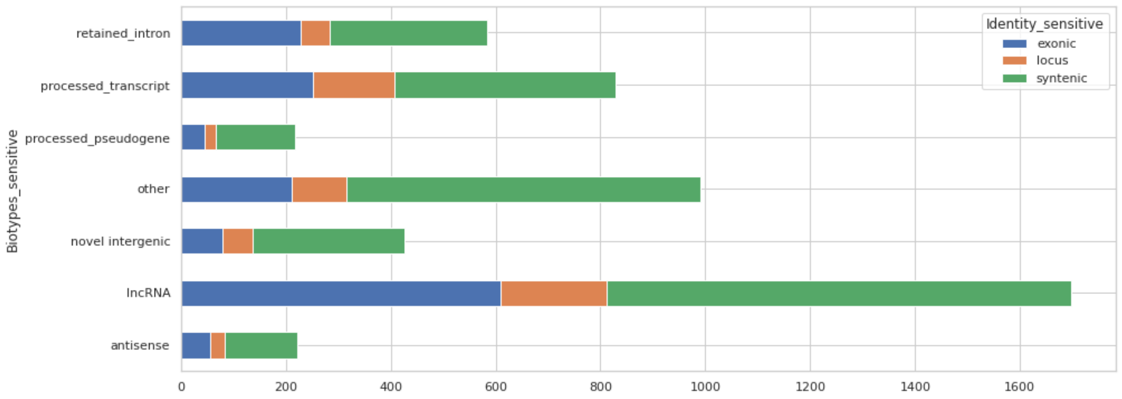

Figure 30. Distributions of transcripts according to conservation type(syntenic/locus/exonic) for dataset based on sensitive approach

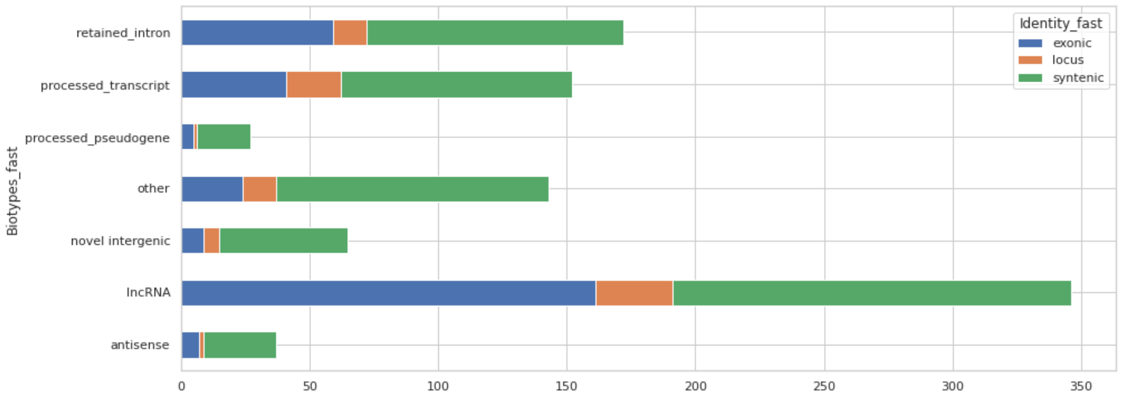

Figure 31. Distributions of transcripts according to conservation type(syntenic/locus/exonic) for dataset based on fast approach

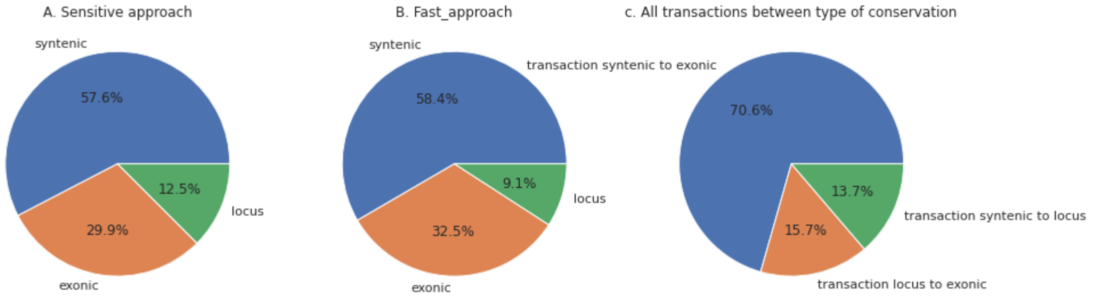

Figure 32. Distributions of transcripts according to conservation type(syntenic/locus/exonic) for dataset based on sensitive approach(**A**), fast approach(**B**) and fraction of transcripts which changed

the conservation type in sensitive approach in comparison with fast(**C**)

3.2.4 Summary

Utilizing a fast approach we detect 942 human transcripts with orthologous counterparts in mice. Using a sensitive approach we were able to detect 4965 human transcripts with orthologs. Data obtained from both strategies(fast and sensitive) differer on 4023 transcripts

[83]:

	Dataset
sensitive_dataset	4051
in both datasets	914
fast_dataset	28

Table 13. Interception of datasets

From 942 human transcripts detected with fast approach 914 were also detected with sensitive approach. The distributions of exonic as well as locus identities between two approaches are close to be equal in relative number, as well as distribution of biotypes. There are some transactions, when for instance transcript pairs showed only syntenic identity when fast approach used, but with sensitive turns into exonic or locus.

[84]:

	transactions
transaction syntenic to exonic	72
transaction locus to exonic	16
transaction syntenic to locus	14

Table 14. All the transactions of transcripts from fast dataset in sensitive

To summing up - we provide 3 different options regarding to sensitivity - first is "fast"("near_fast", "medium_fast" and "far_fast") option which may lose some of conserved lncRNA, performs well on closely related species, but may be done on single server in reasonable time. Another option is to use "sensitive" settings, in that computations take much more time and require proper computational infrastructure, but results will be much more precise. As a trade-off user may use cross-species alignments from UCSC, and get the same sensitivity like in case of "sensitive" settings, disadvantage of that - small number of available species and genome versions.

4 Case study

Here we want to check the conservation of some well known lncRNAs

4.1 Growth arrest-specific 5 RNA (GAS5)

The spliced and poly-adenylated Growth Arrest-Specific 5 (GAS5) RNA was initially identified as a putative tumor suppressor gene due to its accumulation during growth arrest 32 . Sequence comparison between lncRNA-GAS5 exons in humans and mice indicated poor conservation. In contrast, some parts of the introns contained highly conserved regions, which were revealed to be the locus for several small nucleolar RNAs (snoRNAs)

Reference: Evolutionary conservation of long noncoding RNAs; sequence,structure, function

Human transcriptome:

[87] :

	Transcript	Experiment	Gene Name	Description
478	MSTRG.3371.5	hsapiens	GAS5	growth arrest specific 5
479	MSTRG.3371.4	hsapiens	GAS5	growth arrest specific 5
480	MSTRG.3371.7	hsapiens	GAS5	growth arrest specific 5
481	MSTRG.3371.1	hsapiens	GAS5	growth arrest specific 5
482	MSTRG.3371.3	hsapiens	GAS5	growth arrest specific 5
2898	MSTRG.3371.6	hsapiens	GAS5	growth arrest specific 5

Fast approach:

[88] :

	Human	Mouse	alignScore	exonID	locusID	spliceConserved
797	MSTRG.3371.5	MSTRG.7623.2	290760.0	0.36	0.55	5.5
798	MSTRG.3371.4	MSTRG.7623.3	290241.0	0.35	0.58	4.0
799	MSTRG.3371.7	MSTRG.7623.3	290760.0	0.26	0.54	4.0
800	MSTRG.3371.3	MSTRG.7623.4	290241.0	0.30	0.51	6.0
801	MSTRG.3371.6	MSTRG.7623.4	290241.0	0.45	0.59	5.0
802	MSTRG.3371.1	MSTRG.7623.4	45642.0	0.07	0.23	0.5

Sensitive approach:

[89] :

	Human	Mouse	alignScore	exonID	locusID	spliceConserved
3871	MSTRG.3371.5	MSTRG.7623.2	290760.0	0.36	0.55	5.5
3872	MSTRG.3371.4	MSTRG.7623.3	290241.0	0.35	0.58	4.0
3873	MSTRG.3371.7	MSTRG.7623.3	290760.0	0.26	0.54	4.0
3876	MSTRG.3371.3	MSTRG.7623.4	290241.0	0.30	0.51	6.0
3877	MSTRG.3371.6	MSTRG.7623.4	290241.0	0.45	0.59	5.0
3878	MSTRG.3371.1	MSTRG.7623.4	45642.0	0.07	0.23	0.5

Mouse countrparts:

[90] :

	Transcript	Experiment	Gene Name	Description
11078	MSTRG.7623.3	mmusculus	Gas5	growth arrest specific 5
11079	MSTRG.7623.2	mmusculus	Gas5	growth arrest specific 5
11080	MSTRG.7623.4	mmusculus	Gas5	growth arrest specific 5

4.2 MALAT1

The lncRNAs MALAT-1 (metastasis-associated lung adenocarcinoma transcript 1, also known as NEAT2) has been identified to be evolutionary conserved within multiple mammalian species, while no homologues was present in non-mammalian species.

Reference: Evolutionary conservation of long noncoding RNAs; sequence,structure, function

Human transcriptome:

[92] :

	Transcript	Experiment	Gene Name	Description
8345	MSTRG.7876.1	hsapiens	MALAT1	metastasis associated lung adenocarcinoma tran...
8346	MSTRG.7876.3	hsapiens	MALAT1	metastasis associated lung adenocarcinoma tran...
8347	MSTRG.7876.2	hsapiens	MALAT1	metastasis associated lung adenocarcinoma tran...
8348	MSTRG.7876.5	hsapiens	MALAT1	metastasis associated lung adenocarcinoma tran...
8349	MSTRG.7876.4	hsapiens	MALAT1	metastasis associated lung adenocarcinoma tran...
8350	MSTRG.7876.6	hsapiens	MALAT1	metastasis associated lung adenocarcinoma tran...

Fast approach (no counterparts detected)

Sensitive approach:

[94]:

	Human	Mouse	alignScore	exonID	locusID	spliceConserved
3245	MSTRG.7876.1	MSTRG.61414.1	455430.0	0.60	0.75	0.0
3246	MSTRG.7876.3	MSTRG.61414.1	455430.0	0.61	0.75	0.0
3247	MSTRG.7876.2	MSTRG.61414.1	453259.0	0.68	0.69	0.0
3248	MSTRG.7876.5	MSTRG.61414.1	446951.0	0.75	0.77	0.0
3249	MSTRG.7876.4	MSTRG.61414.1	449342.0	0.58	0.62	0.0
3250	MSTRG.7876.6	MSTRG.61414.1	445730.0	0.79	0.77	1.0

Mouse countrparts:

[95]:

	Transcript	Experiment	Gene Name	Description
16882	MSTRG.61414.1	mmusculus	Malat1	metastasis associated lung adenocarcinoma tran...

4.3 HOTAIRM1

This non-coding locus is located in the HOX gene cluster. Transcription of this locus is induced by retinoic acid, and transcripts likely function in regulation of myelopoiesis through transcriptional activation of several genes in the HOXA cluster, in addition to several beta-2 integrins. According to Rfam this gene part of HOXA conserved region.

Human transcriptome:

[97]:

	Transcript	Experiment	Gene Name	Description
8216	MSTRG.42449.1	hsapiens	HOTAIRM1	HOXA transcript antisense RNA, myeloid-specific 1

Fast approach:

[98]:

	Human	Mouse	alignScore	exonID	locusID	spliceConserved
0	MSTRG.42449.1	MSTRG.100138.1	1394660.0	0.35	0.63	1.0

Sensitive approach:

[99]:

	Human	Mouse	alignScore	exonID	locusID	spliceConserved
3	MSTRG.42449.1	MSTRG.100138.1	1394660.0	0.35	0.63	1.0

Mouse countrparts:

[100]:

	Transcript	Experiment	Gene Name	Description
21177	MSTRG.100138.1	mmusculus	Hotairm1	Hoxa transcript antisense RNA, myeloid-specific 1

4.4 The X inactive specific transcript

One of few lncRNAs which has been extensively characterized on both the functional and structural level, is the X inactive specific transcript (Xist). Xist is a ~17kb lncRNA essential for mammalian X chromosome inactivation

Reference Evolutionary conservation of long noncoding RNAs; sequence,structure, function

Human transcriptome:

[102]:

	Transcript	Experiment	Gene Name	Description
6806	MSTRG.50430.7	hsapiens	XIST	X inactive specific transcript

Fast approach (no counterparts detected)

Sensitive approach:

[104]:

	Human	Mouse	alignScore	exonID	locusID	spliceConserved
1192	MSTRG.50430.7	MSTRG.130357.1	131842.0	0.48	0.43	0.5

Mouse countrparts:

[105]:

	Transcript	Experiment	Gene Name	Description
12292	MSTRG.130357.1	mmusculus	Xist	inactive X specific transcripts

4.5 CHD2 adjacent, suppressive regulatory RNA(CHASERR)

The human lncRNA CHASERR (CHD2 adjacent, suppressive regulatory RNA) is found upstream of Chd2 (chromodomain helicase DNA binding protein 2) in both mice and humans and exhibits further conservation throughout the vertebrate lineage. Sequentially and syntenically conserved between humans and mice. Extron–intron structure and some sequence conserved across vertebrates.

Reference: Long non-coding RNAs in development and disease: conservation to mechanisms

Human transcriptome:

[107]:

	Transcript	Experiment	Gene Name	Description
3460	MSTRG.16679.1	hsapiens	LINC01578	long intergenic non-protein coding RNA 1578

Fast approach (no counterparts detected)

Sensitive approach:

[109]:

	Human	Mouse	alignScore	exonID	locusID	spliceConserved
436	MSTRG.16679.1	MSTRG.109096.13	675317.0	0.48	0.59	2.5

Mouse countrparts:

[110]:

	Transcript	Experiment	Gene Name	Description
1447	MSTRG.109096.13	mmusculus	1810026B05Rik	RIKEN cDNA 1810026B05 gene

4.6 CEROX1 cytoplasmic endogenous regulator of oxidative phosphorylation 1

For the majority of conserved lncRNAs, only a moderate level of sequence identity is observed 17. This is demonstrated by Cerox1, an intergenic, bi-directional lncRNA found in mice (Table 1). Cerox1 regulates the abundance of the mitochondrial complex 1 transcript by acting as a miRNA decoy, therefore modulating its activity. Although Cerox1 is conserved at the sequence level and is syntenically homologous with a human homologue, the level of conservation drops rapidly in more distant species. Across eutherian mammals, conservation is found only in the second exon of Cerox1

Reference: Long non-coding RNAs in development and disease: conservation to mechanisms

Human transcriptome:

[112]:

	Transcript	Experiment	Gene Name	Description
4001	MSTRG.16879.1	hsapiens	CEROX1	cytoplasmic endogenous regulator of oxidative ...

Fast approach (no counterparts detected)

Sensitive approach:

[114]:

	Human	Mouse	alignScore	exonID	locusID	spliceConserved
2850	MSTRG.16879.1	MSTRG.52730.1	66786.0	0.37	0.15	0.0

Mouse countrparts:

[115]:

	Transcript	Experiment	Gene Name	Description
8064	MSTRG.52730.1	mmusculus	Cerox1	cytoplasmic endogenous regulator of oxidative ...

4.7 LINC00473 (PDE10A) (primate specific)

LINC00473 (NCBI gene record; description: long intergenic non-protein coding RNA 473) is an external reference matched to Gene ENSG00000112541)

Primate-specific lncRNA, LINC00473, that regulates human thermogenic adipocyte metabolism, specifically fatty acid flux and respiration, thereby advancing understanding of lncRNA regulation in adipocytes and obesity.

Reference: A functional non-conserved long non-coding RNA in human adipose tissue

Human counterparts:

[117]:

	Transcript	Experiment	Gene Name	Description
1559	MSTRG.41765.1	hsapiens	PDE10A	phosphodiesterase 10A

Not found by both fast and sensitive methods

4.8 FTX (FTX transcript, XIST regulator)

The Ftx transcript is a conserved functional lncRNA encoded within the X-inactivation center (Xic). Ftx encodes 4 microRNAs in its introns [7]. Intron 12 encodes 1 cluster of 2 microRNAs (miR-374b and miR-421), which is well conserved in different mammalian species. Intron b encodes 1 related cluster of 2 microRNAs (miR-374a and miR-545), which is absent in mouse and rat due to mutational changes

Reference: The miR-545/374a Cluster Encoded in the Ftx lncRNA is Overexpressed in HBV-Related Hepatocellular Carcinoma and Promotes Tumorigenesis and Tumor Progression

Human transcriptome:

[119]:

	Transcript	Experiment	Gene Name	Description
274	MSTRG.50527.8	hsapiens	FTX	FTX transcript, XIST regulator
277	MSTRG.50527.6	hsapiens	FTX	FTX transcript, XIST regulator
278	MSTRG.50527.3	hsapiens	FTX	FTX transcript, XIST regulator
1602	MSTRG.50527.4	hsapiens	FTX	FTX transcript, XIST regulator
2295	MSTRG.50527.19	hsapiens	FTX	FTX transcript, XIST regulator
2297	MSTRG.50527.17	hsapiens	FTX	FTX transcript, XIST regulator
2300	MSTRG.50527.13	hsapiens	FTX	FTX transcript, XIST regulator
2628	MSTRG.50527.1	hsapiens	FTX	FTX transcript, XIST regulator
2776	MSTRG.50527.2	hsapiens	FTX	FTX transcript, XIST regulator
3373	MSTRG.50527.16	hsapiens	FTX	FTX transcript, XIST regulator
7684	MSTRG.50537.1	hsapiens	FTX	FTX transcript, XIST regulator
7856	MSTRG.50527.20	hsapiens	FTX	FTX transcript, XIST regulator
8302	MSTRG.50450.1	hsapiens	FTX	FTX transcript, XIST regulator
9222	MSTRG.50449.1	hsapiens	FTX	FTX transcript, XIST regulator
9308	MSTRG.50528.2	hsapiens	FTX	FTX transcript, XIST regulator

Fast approach (no counterparts detected)

Sensitive approach:

[120]:

	Human	Mouse	alignScore	exonID	locusID	spliceConserved
1194	MSTRG.50527.3	MSTRG.130393.1	97903.0	0.00	0.00	0.0
1196	MSTRG.50527.4	MSTRG.130408.3	123964.0	0.03	0.08	0.0
1197	MSTRG.50527.6	MSTRG.130408.3	72347.0	0.07	0.19	1.5
1198	MSTRG.50527.2	MSTRG.130408.3	123964.0	0.01	0.08	0.0
1199	MSTRG.50527.19	MSTRG.130408.4	56325.0	0.09	0.40	0.0
1200	MSTRG.50527.17	MSTRG.130408.4	69345.0	0.11	0.28	1.5
1201	MSTRG.50527.16	MSTRG.130408.4	69345.0	0.07	0.27	1.5
1202	MSTRG.50527.13	MSTRG.130408.4	69345.0	0.04	0.26	1.5
1203	MSTRG.50527.20	MSTRG.130408.4	56325.0	0.09	0.38	0.0
1204	MSTRG.50527.8	MSTRG.130408.4	69345.0	0.07	0.14	1.5

Mouse counterparts:

[121]:

	Transcript	Experiment	Gene Name	Description
1958	MSTRG.130408.4	mmusculus	Ftx	Ftx transcript, Xist regulator (non-protein co...
1962	MSTRG.130408.3	mmusculus	Ftx	Ftx transcript, Xist regulator (non-protein co...
3847	MSTRG.130393.1	mmusculus	NaN	NaN

4.9 JPX (JPX, XIST activator)

Long noncoding RNAs (lncRNAs) have been identified in all eukaryotes and are most abundant in the human genome. However, the functional importance and mechanisms of action for human lncRNAs are largely unknown. Using comparative sequence, structural, and functional analyses, we characterize the evolution and molecular function of human lncRNA JPX. Was found that human JPX and its mouse homolog, lncRNA Jpx, have deep divergence in their nucleotide sequences and RNA secondary structures.

Reference: Functional Conservation of LncRNA JPX Despite Sequence and Structural Divergence

Human transcriptome:

[123]:

	Transcript	Experiment	Gene Name	Description
2477	MSTRG.50448.5	hsapiens	JPX	JPX transcript, XIST activator
2498	MSTRG.50448.9	hsapiens	JPX	JPX transcript, XIST activator
7493	MSTRG.50448.7	hsapiens	JPX	JPX transcript, XIST activator
7494	MSTRG.50448.6	hsapiens	JPX	JPX transcript, XIST activator
7495	MSTRG.50448.1	hsapiens	JPX	JPX transcript, XIST activator
7496	MSTRG.50448.3	hsapiens	JPX	JPX transcript, XIST activator
7497	MSTRG.50448.2	hsapiens	JPX	JPX transcript, XIST activator

Fast approach (no conterparts detected)

Sensitive approach:

[124]:

	Human	Mouse	alignScore	exonID	locusID	spliceConserved
1188	MSTRG.50448.5	MSTRG.130339.2	52922.0	0.10	0.55	0.0
1189	MSTRG.50448.7	MSTRG.130339.2	52922.0	0.04	0.54	0.0
1190	MSTRG.50448.3	MSTRG.130339.2	52922.0	0.03	0.14	0.0

Mouse counterpart:

[125]:

	Transcript	Experiment	Gene Name	Description
20869	MSTRG.130339.2	mmusculus	Jpx	Jpx transcript, Xist activator (non-protein co...

4.10 RMST (Rhabdomyosarcoma 2 Associated Transcript)

This gene produces a long non-coding RNA that functions in neurogenesis by aiding in the association of Sox2 transcription factor to its target promoters. [provided by RefSeq, Dec 2017] RMST orthologs from human to frog are well conserved at important gene regulatory regions, including their promoter regions, first exons, and splice sites

Reference: The Long Noncoding RNA RMST Interacts with SOX2 to Regulate Neurogenesis

Human transcriptome:

[127]:

	Transcript	Experiment	Gene Name	Description
9713	MSTRG.11371.3	hsapiens	RMST	rhabdomyosarcoma 2 associated transcript

Fast approach (counterpart not found)

Sensitive approach:

[128]:

	Human	Mouse	alignScore	exonID	locusID	spliceConserved
1336	MSTRG.11371.3	MSTRG.15054.1	1411457.0	0.58	0.29	1.5

Mouse counterpart:

[129]:

	Transcript	Experiment	Gene Name	Description
24242	MSTRG.15054.1	mmusculus	Rmst	rhabdomyosarcoma 2 associated transcript (non-...

4.11 ADAMTS9-AS2

ADAMTS9-AS2 is well conservative among different species, indicating its potential of regulatory functions in different species.

Reference: LncRNA ADAMTS9-AS2 inhibits cell proliferation and decreases chemoresistance in clear cell renal cell carcinoma via the miR-27a-3p/FOXO1 axis

Human transcriptome:

[131]:

	Transcript	Experiment	Gene Name	Description
3609	MSTRG.31593.1	hsapiens	ADAMTS9-AS2	ADAMTS9 antisense RNA 2
5863	MSTRG.31590.6	hsapiens	ADAMTS9-AS2	ADAMTS9 antisense RNA 2
5866	MSTRG.31590.2	hsapiens	ADAMTS9-AS2	ADAMTS9 antisense RNA 2
5867	MSTRG.31590.1	hsapiens	ADAMTS9-AS2	ADAMTS9 antisense RNA 2
8382	MSTRG.31590.5	hsapiens	ADAMTS9-AS2	ADAMTS9 antisense RNA 2

Fast approach (counterpart not found)

Sensitive approach:

[132]:

	Human	Mouse	alignScore	exonID	locusID	spliceConserved
69	MSTRG.31590.5	MSTRG.102067.1	98261.0	0.00	0.00	0.0
70	MSTRG.31590.6	MSTRG.102112.1	173852.0	0.00	0.00	0.0
71	MSTRG.31590.2	MSTRG.102124.2	173371.0	0.06	0.00	0.0
72	MSTRG.31590.1	MSTRG.102124.2	358274.0	0.00	0.01	0.0

Mouse counterparts:

[133]:

	Transcript	Experiment	Gene Name	Description
3145	MSTRG.102067.1	mmusculus	9530026P05Rik	RIKEN cDNA 9530026P05 gene
8370	MSTRG.102124.2	mmusculus	9530026P05Rik	RIKEN cDNA 9530026P05 gene
11000	MSTRG.102112.1	mmusculus	9530026P05Rik	RIKEN cDNA 9530026P05 gene
